# Supplementary material for: Predicting corporate management performance using AI: Incorporating CEO strategy insights from sustainable management reports
Source: PLoS One. 2026 May 6;21(5):e0347140. doi: 10.1371/journal.pone.0347140 (PMC13148710; doi:10.1371/journal.pone.0347140)

PLOS ONE

Predicting Corporate Management Performance Using AI: Incorporating CEO Strategy Insights from Sustainable Management Reports

--Manuscript Draft--

**Manuscript Number:**	PONE-D-24-38231
**Article Type:**	Research Article
**Full Title:**	Predicting Corporate Management Performance Using AI: Incorporating CEO Strategy Insights from Sustainable Management Reports
**Short Title:**	Predicting Corporate Management Performance Using AI
**Corresponding Author:**	Yubing wei<br>Semyung University<br>Jecheon-si, Chungcheongbuk-do KOREA, REPUBLIC OF
**Keywords:**	Firm management performance, Sustainable management report, Text mining, Corporate management strategy, SBSC framework, Machine learning, Deep learnin
**Abstract:**	This research employed AI based on machine learning and deep learning classification techniques to forecast corporate management performance. In this paper, researchers used various machine learning and deep learning classifiers, including KNN, SVM, GBM, CatBoost, GAN, RNN, LSTM, and Transformer. The corporate management performance prediction model developed in this study uses financial variables that have a significant relationship with corporate management performance and management strategy based on prior research on these topics. Specifically, this study compared the performance of the basic prediction model, including only the company's financial variables, and the model upgraded by including management strategy information based on the SBSC (Sustainable Balanced Scorecard) framework in the company's financial variables. For the information on the company's management strategy, the contents of the CEO message of the sustainable management report were extracted through the text mining technique. The empirical analysis results of this paper are as follows. First, we found that the predictive model's performance incorporating management strategy information extracted from the company's sustainability management report was superior to that of the predictive model using financial variables only. Second, Transformer's performance best predicted the company's management performance among the eight classifiers. Next, LSTM, RNN, GAN, GBM, CatBoost, SVM, and KNN performed excellently. The research methodology presented in this study is expected to contribute academically to expanding the scope of research in predicting corporate management performance. In addition, this study is expected to provide useful information for decision-making by many corporate stakeholders, such as managers and investors, by presenting a high-performance corporate management performance prediction model in practice.
**Order of Authors:**	Xiao Wang
	Feng Sun
	Yong Ki Kim
	Hyungjoon Kim
	Wonho Song
	Yubing wei
**Additional Information:**	
**Question**	**Response**
**Financial Disclosure**<br><br>Enter a financial disclosure statement that describes the sources of funding for the work included in this submission. Review	The author(s) received no specific funding for this work.

Powered by Editorial Manager® and ProduXion Manager® from Aries Systems Corporation

the submission guidelines for detailed requirements. View published research articles from *PLOS ONE* for specific examples.<br><br>This statement is required for submission and **will appear in the published article** if the submission is accepted. Please make sure it is accurate.<br><br>**Funded studies**<br>Enter a statement with the following details:<br>• Initials of the authors who received each award<br>• Grant numbers awarded to each author<br>• The full name of each funder<br>• URL of each funder website<br>• Did the sponsors or funders play any role in the study design, data collection and analysis, decision to publish, or preparation of the manuscript?<br><br>Did you receive funding for this work?	
**Competing Interests**<br><br>Use the instructions below to enter a competing interest statement for this submission. On behalf of all authors, disclose any competing interests that could be perceived to bias this work—acknowledging all financial support and any other relevant financial or non-financial competing interests.<br><br>This statement is required for submission and **will appear in the published article** if the submission is accepted. Please make sure it is accurate and that any funding sources listed in your Funding Information later in the submission form are also declared in your Financial Disclosure statement.<br><br>View published research articles from *PLOS ONE* for specific examples.	The authors have declared that no competing interests exist.

Powered by Editorial Manager® and ProduXion Manager® from Aries Systems Corporation

**NO authors have competing interests**<br><br>Enter: *The authors have declared that no competing interests exist.*<br><br>**Authors with competing interests**<br><br>Enter competing interest details beginning with this statement:<br><br>*I have read the journal's policy and the authors of this manuscript have the following competing interests: [insert competing interests here]*<br><br>* typeset	
**Ethics Statement**<br><br>Enter an ethics statement for this submission. This statement is required if the study involved:<br><br>• Human participants<br>• Human specimens or tissue<br>• Vertebrate animals or cephalopods<br>• Vertebrate embryos or tissues<br>• Field research<br><br>Write "N/A" if the submission does not require an ethics statement.<br><br>General guidance is provided below. Consult the submission guidelines for detailed instructions. **Make sure that all information entered here is included in the Methods section of the manuscript.**	N/A

**Format for specific study types**<br><br>**Human Subject Research (involving human participants and/or tissue)**<br>• Give the name of the institutional review board or ethics committee that approved the study<br>• Include the approval number and/or a statement indicating approval of this research<br>• Indicate the form of consent obtained (written/oral) or the reason that consent was not obtained (e.g. the data were analyzed anonymously)<br><br>**Animal Research (involving vertebrate animals, embryos or tissues)**<br>• Provide the name of the Institutional Animal Care and Use Committee (IACUC) or other relevant ethics board that reviewed the study protocol, and indicate whether they approved this research or granted a formal waiver of ethical approval<br>• Include an approval number if one was obtained<br>• If the study involved *non-human primates*, add *additional details* about animal welfare and steps taken to ameliorate suffering<br>• If anesthesia, euthanasia, or any kind of animal sacrifice is part of the study, include briefly which substances and/or methods were applied<br><br>**Field Research**<br><br>Include the following details if this study involves the collection of plant, animal, or other materials from a natural setting:<br>• Field permit number<br>• Name of the institution or relevant body that granted permission	
**Data Availability**<br><br>Authors are required to make all data underlying the findings described fully available, without restriction, and from the time of publication. PLOS allows rare exceptions to address legal and ethical concerns. See the PLOS Data Policy and FAQ for detailed information.	Yes - all data are fully available without restriction

A Data Availability Statement describing where the data can be found is required at submission. Your answers to this question constitute the Data Availability Statement and **will be published in the article**, if accepted.<br><br>**Important:** Stating 'data available on request from the author' is not sufficient. If your data are only available upon request, select 'No' for the first question and explain your exceptional situation in the text box.<br><br>Do the authors confirm that all data underlying the findings described in their manuscript are fully available without restriction?	
**Describe where the data may be found in full sentences. If you are copying our sample text, replace any instances of XXX with the appropriate details.**<br><br>• If the data are **held or will be held in a public repository**, include URLs, accession numbers or DOIs. If this information will only be available after acceptance, indicate this by ticking the box below. For example: *All XXX files are available from the XXX database (accession number(s) XXX, XXX.)*.<br>• If the data are all contained **within the manuscript and/or Supporting Information files**, enter the following: *All relevant data are within the manuscript and its Supporting Information files.*<br>• If neither of these applies but you are able to provide **details of access elsewhere**, with or without limitations, please do so. For example:<br><br>*Data cannot be shared publicly because of [XXX]. Data are available from the XXX Institutional Data Access / Ethics Committee (contact via XXX) for researchers who meet the criteria for access to confidential data.*<br><br>*The data underlying the results presented in the study are available from (include the name of the third party*	The data used in this study are available from the following sources:<br>1. sustainability management report provided by the ESG Portal Site (https://esg.krx.co.kr)<br>2. NICE Credit Rating Information's Value Search (http://www.nicerating.com)

and contact information or URL).<br>• This text is appropriate if the data are owned by a third party and authors do not have permission to share the data.<br><br>* typeset	
Additional data availability information:	Tick here if the URLs/accession numbers/DOIs will be available only after acceptance of the manuscript for publication so that we can ensure their inclusion before publication.

Manuscript
Click here to access/download;Manuscript;20240902
PlosOneManuscript.docx

Predicting Corporate Management Performance Using AI: Incorporating CEO Strategy Insights from Sustainable Management Reports

Xiao Wang[1], Feng Sun[2], Yong Ki Kim[3], Hyungjoon Kim[4], WonHo Song[5] and Yubing Wei[6]*

[1]School of Business Administration, Binzhou Polytechnic, Binzhou City, Shandong Province, China;

[2]School of Humanities, Binzhou Polytechnic, Bincheng District, Binzhou City, Shandong Province, China;

[3]Department of Taxation, Accounting, and Finance, Sejong Cyber University, Gwangjingu, Seoul, Republic of Korea;

[4]Department of Computer Engineering, Changwon National University, Uichanggu, Changwonsi, Gyeongsangnamdo, Republic of Korea;

[5]Department of Computer Engineering, Changwon National University, Uichanggu, Changwonsi, Gyeongsangnamdo, Republic of Korea;

[6]Department of Business Administration, Semyung University, Jecheonsi, Chungcheongbukdo, Korea, 27136;

*Correspondence: weiwei992@naver.com(YW)

Abstract

This research employed AI based on machine learning and deep learning classification techniques to forecast corporate management performance. In this paper, researchers used various machine learning and deep learning classifiers, including KNN, SVM, GBM, CatBoost, GAN, RNN, LSTM, and Transformer. The corporate management performance

prediction model developed in this study uses financial variables that have a significant relationship with corporate management performance and management strategy based on prior research on these topics. Specifically, this study compared the performance of the basic prediction model, including only the company's financial variables, and the model upgraded by including management strategy information based on the SBSC (Sustainable Balanced Scorecard) framework in the company's financial variables. For the information on the company's management strategy, the contents of the CEO message of the sustainable management report were extracted through the text mining technique. The empirical analysis results of this paper are as follows. First, we found that the predictive model's performance incorporating management strategy information extracted from the company's sustainability management report was superior to that of the predictive model using financial variables only. Second, Transformer's performance best predicted the company's management performance among the eight classifiers. Next, LSTM, RNN, GAN, GBM, CatBoost, SVM, and KNN performed excellently. The research methodology presented in this study is expected to contribute academically to expanding the scope of research in predicting corporate management performance. In addition, this study is expected to provide useful information for decision-making by many corporate stakeholders, such as managers and investors, by presenting a high-performance corporate management performance prediction model in practice.

Introduction

The global management trend has recently focused on corporate ESG activities [1]. Corporate management strategies are also naturally established with a focus on ESG activities [2]. Amid

these changes in the business environment, predicting corporate management performance in advance is very important, academically and practically.

The sustainability management report contains information about the company's ESG activities, and the CEO message, in particular, contains information about the company's management strategy [3, 4]. Although competition strategies can be identified in corporate reports such as business reports and management diagnosis opinions (MD&A), this study attempts to understand the management strategies of companies from sustainable management reports because recent management trends are interested in ESG activities. A common CEO message at the beginning of the sustainability management report is implicitly well expressed about the company's vision, goals, and management strategies [5, 6]. Therefore, this study uses text mining techniques to collect nonfinancial information from the CEO of the sustainability management report and reflects it in the management performance prediction model.

Previous studies on predicting corporate management performance were designed mainly based on corporate financial data [7, 8]. However, corporate information is not composed only of financial data; non-financial information contains many important contents. This study attempts to improve predictive performance by extracting the contents of the corporate management strategy from the CEO message of the sustainable management report, classifying them by the SBSC (Sustainable Balanced Scorecard) framework, and reflecting them in the corporate management performance prediction model.

A company's management strategy is important information that determines its future [9]. Predicting the company's management performance is judged to be an accurate way to do this. In this study, the management strategy of a company is included in the management

performance prediction model by identifying which parts of the management strategy are emphasized according to the SBSC framework. An accurate prediction model can provide useful information when making decisions for a company's managers, investors, and various stakeholders.

Predictive technology has increased thanks to the rapid development of artificial intelligence-based machine learning and deep learning technologies [10]. In addition, machine learning and deep learning technologies can effectively analyze various data. Suppose the company's financial data and the management strategy information of the non-financial sustainability management report CEO message are refined by text mining techniques and included in the model. In that case, it is expected to show excellent predictive performance.

In other words, this paper aims to develop an AI-based prediction model that can more accurately predict a company's management performance by reflecting the information contained in the CEO message of the sustainability management report. The management performance prediction model that reflects management strategy information, including corporate financial data and ESG activities, is expected to perform higher than the existing management performance prediction model. In addition, the management performance prediction model to be developed in this paper is systematically and logically designed by using financial variables that have already been verified in previous studies in accounting.

The research results of this paper are expected to present a new research methodology in the academic management performance prediction research field. They are expected to contribute to developing convergence research between business administration and computer engineering. In practice, the corporate management performance prediction model presented in this study is expected to help various company stakeholders.

The differences between this study and previous studies are as follows. First, existing management performance prediction studies predicted a company's management performance using all available corporate financial data without being based on accounting theory. Still, based on previous accounting studies, this study designs a model using financial variables related to management performance. Second, as the author knows, the world's first CEO message of a sustainability management report is collected and refined by text mining techniques and included in the model and financial data, thereby improving predictive performance. Third, this study uses machine learning and deep learnin[illegible]chniques to compare and analyze the analysis results by eight classes. In other words, this paper's management performance prediction results are customer-oriented and highly reliable. In particular, by comparing the performance of a model using only corporate financial information and a model adding strategic information, which is non-financial information, it is found that the predictive performance of the model adding corporate strategic information is better.

This paper has academic value because it distinguishes itself from previous studies. Its results can present data necessary for establishing a management strategy that considers a company's ESG activities and provides meaningful information for sustainable growth.

Data and methods

Data

The company's sustainability management report, made publicly available, contains crucial details about its ESG activities, vision, and strategic objectives [4]. Notably, the CEO’s

message within this report subtly conveys the company’s strategic direction [3].

In this study, text data from the sustainability management report was obtained using text mining techniques and then processed using Natural Language Processing (NLP) [11]. Text normalization was employed during the text preprocessing phase to eliminate unnecessary punctuation marks and special characters. Stop Word Removal was then applied to filter out irrelevant or redundant words [12]. Tokenization followed, breaking down the text into words, sentences, or other meaningful units [13]. The processed texts were subsequently represented as vectors using the Bag of Words (BoW) model [14]. Key terms frequently appearing in the sustainability management report were identified by calculating TF-IDF (Term Frequency-Inverse Document Frequency) values, with higher values indicating significant keywords [15].

Keywords were selected only for words with a TF-IDF value of 1.5 or higher. Then, according to the SBSC framework, the keywords extracted from the CEO message of the sustainability management report were classified into financial, customer, internal process, learning and growth, and sustainability categories [16, 17]. The criteria for classifying management strategies emphasized by companies were based on which point of view the keywords extracted from the CEO message of each company's sustainability management report, that is, words with a high TF-IDF, were most classified among finance, customers, internal processes, learning and growth, and sustainability perspective.

For example, if there are five keywords corresponding to a finance perspective, three keywords corresponding to a customer perspective, and two corresponding to an internal process perspective, the company's management strategy was judged to emphasize a finance perspective the most, so one was assigned to the finance variable, and 0 was assigned to the

remaining SBSC framework variables. Through this method, this study quantified the management strategy information that companies emphasize the most.

The management strategy data used in this study was taken directly from a sustainability management report provided by the ESG Portal Site (https://esg.krx.co.kr). The CEO message in this report summarizes each company's annual vision, goals, and business outlook.

Financial data for this research was obtained from NICE Credit Rating In-formation’s Value Search (http://www.nicerating.com). The analysis spanned from 2016 to 2023, focusing on companies listed on KOSPI and KOSDAQ. The initial dataset included 16,343 companies listed on KOSPI and KOSDAQ between 2016 and 2023, excluding those in the financial sector. However, companies that did not close their accounts in December 385, those without available financial data 993, and companies with capital erosion due to sustained losses 35 were excluded. Additionally, 8,455 companies that did not disclose ESG ratings from the Korea ESG Standards Institute were omitted, as ESG rating information was crucial for the prediction model. Furthermore, since the study relied on management strategy information derived from the CEO's message in the sustainability management report, 5,604 companies that did not disclose such a re-port were also excluded. As a result, the final sample used in the empirical analysis comprised 871 companies. Table 1 below shows the sample information utilized in this empirical analysis.

Table 1. Corporate sample selection

Sample selection	Sample number

Listed companies excluding the financial industry from 2016 to 2023	16,343
Excluding companies for December settlement	-385
Excluding enterprises that erode capital	-35
Exclude companies without corporate financial data	-993
Excluding companies that do not disclose ESG ratings by the Korea ESG Standards Institute	-8,455
Excluding companies that have not disclosed sustainability management reports	-5,604
Final sample from 2016 to 2023	871

Table 2 summarizes the variables used in this paper's research model. ROE measures corporate management performance, and the research focuses on developing machine learning and deep learning models to predict the firm performance. The Finance variable is a dummy variable; if the most repeated keywords are related to finance in the Sustainable Management Report's CEO message, it is 1; otherwise, it is 0. Similarly, if the most repeated keywords in the Sustainable Management Report's CEO message are related to customers, a dummy variable is 1; otherwise, it is 0. The internal process is a dummy variable; if the most repeated keywords are related to the internal process in the Sustainable Management Report's CEO message, it is 1; otherwise, it is 0. The Learning & Growth is a dummy variable; if the most repeated keywords are related to the employee's education for learning and growth in the Sustainable Management Report's CEO message, it is 1; otherwise, it is 0. Lastly, Sustainability is a dummy variable; if the most repeated keywords in the Sustainable Management Report's CEO message are related to the environment, donation, sustainable growth, and so on, it is 1; otherwise, it is 0. The meaning of the most frequently used keywords in this study is that there are relatively many keywords with high TF-IDF values. The management strategy variables used in this study are designed to capture specific aspects

of a company's management strategy delivered through the CEO's message in the sustainability report concerning previous studies [3].

Table 2. Variables definition

Variables	Definition
**ROE**	Return on equity = Net income / Total capital
**Financial**	Dummy variable, if the most keywords for finance in the Sustainable Management Report's CEO message is 1, otherwise 0
**Customer**	Dummy variable, if the most keywords for customers in the Sustainable Management Report's CEO message is 1, otherwise 0
**Internal process**	Dummy variable, if the most keywords for Internal process in the Sustainable Management Report's CEO message is 1, otherwise 0
**Learning & Growth**	Dummy variable, if the most keywords for Learning & Growth in the Sustainable Management Report's CEO message is 1, otherwise 0
**Sustainability**	Dummy variable, if the most keywords for sustainability in the Sustainable Management Report's CEO message is 1, otherwise 0
**SIZE**	Natural logarithmic value of total assets
**Leverage**	Debt ratio = Total liabilities / Total assets
**Current**	Current Ratio = Current Liabilities / Current Assets
**Growth**	The rate of increase/decrease in sales
**Foreigner**	Foreign shareholders' equity ratio
**Largest**	The largest shareholder's equity ratio
**BIG4**	If the accounting firm that conducted the external audit is a major accounting firm corresponding to BIG4, a dummy variable that means 1 or 0
**ESG**	Scores based on the ESG rating of companies disclosed by the Korea ESG Standards Institute (A+=6, A=5, B+=4, B=3, C=2, D=1)

Table 3 provides the descriptive statistics for the variables used in the empirical analysis. The mean value of ROE, which measures corporate management performance, is 0.065, with a standard deviation of 0.136. The Financial variable, indicating the company's financial focus in its management strategy, has a mean of 0.194 and a standard deviation of 0.396. The Customer variable, which emphasizes customer-centric management strategies, shows a mean value of 0.233 with a standard deviation of 0.423. The Internal variable, which focuses on internal processes within the company's management strategy, has an average value of 0.219 and a standard deviation of 0.414. The Learning & Growth variable, reflecting the company's focus on learning and growth strategies, averages 0.233 with a standard deviation of 0.423. Lastly, the Sustainability variable, representing the company's emphasis on sustainability initiatives such as social responsibility and environmental protection, has a mean value of 0.215 and a standard deviation of 0.411. These statistics summarize the central tendencies and variability of the key variables used in the analysis.

Table 3 Descriptive statistics

Variables	N	Mean	Std	Min	Q1	Median	Q3	Max
**ROE**	871	0.065	0.136	-0.831	0.018	0.061	0.117	0.561
**Finance**	871	0.194	0.396	0.000	0.000	0.000	0.000	1.000
**Customer**	871	0.233	0.423	0.000	0.000	0.000	0.000	1.000
**Internal**	871	0.219	0.414	0.000	0.000	0.000	0.000	1.000
**Learning & Growth**	871	0.233	0.423	0.000	0.000	0.000	0.000	1.000
**Sustainability**	871	0.215	0.411	0.000	0.000	0.000	0.000	1.000

**SIZE**	871	28.876	1.335	25.016	27.961	28.948	30.056	30.604
**Leverage**	871	0.432	0.199	0.027	0.266	0.445	0.579	0.875
**Current**	871	2.056	3.637	0.195	0.854	1.247	1.965	32.472
**Growth**	871	0.078	0.328	-0.701	-0.050	0.041	0.154	2.111
**Foreigner**	871	0.199	0.161	0.002	0.077	0.152	0.271	1.000
**Largest**	871	0.408	0.162	0.064	0.300	0.387	0.511	0.917
**BIG4**	871	0.897	0.305	0.000	1.000	1.000	1.000	1.000
**ESG**	871	4.423	0.954	1.000	4.000	5.000	5.000	6.000

Prediction models

This study draws on previous research on corporate management performance to construct a model for predicting such performance. The key variables in the standard model are outlined below.

SIZE variable represents a company's size and is calculated by taking the natural logarithm of its total assets. Due to their scale, larger firms typically demonstrate more stable management performance [18]. Leverage variable indicates the ratio of a company's debt to its total assets. Generally, a higher debt ratio is associated with a weaker financial position and more volatile management performance [19]. Current represents a firm’s liquidity ratio, and this variable indicates financial stability. A company’s liquidity can significantly impact its management strategy and overall performance [20]. Growth refers to the company’s sales growth rate. Higher sales growth is often linked to improved management performance, leading to greater profitability [21]. Foreigner variable captures the proportion of shares held by foreign shareholders. A higher percentage of foreign ownership often leads to better monitoring, contributing to a more transparent governance structure that can enhance management

performance [22]. Largest variable represents the ownership percentage of the largest shareholder. While higher ownership by the largest shareholder can lead to more effective corporate management, some studies suggest that it can also result in poorer corporate governance, negatively impacting management [23, 24]. BIG4 is a dummy variable indicating whether a major accounting firm audited the company. Audits by prominent firms are often linked to larger company size and improved management performance [25]. ESG variable represents the company's ESG (Environmental, Social, and Governance) rating, as disclosed by the Korea ESG Standards Institute [26]. This study quantifies ESG grades on a scale from A+ (6) to D (1). A higher ESG rating generally correlates with better management performance, as it enhances the company's image and, over the long term, is likely to sustain or improve management outcomes.

This research compares the predictive performance of a standard model with an enhanced model that incorporates management strategy information derived from sustainability management reports. The two corporate management performance prediction models are expressed as functions in (1) and (2).

[The standard model for forecasting firm management performance]

$$F(SIZE, Leverage, Current, Growth, Foreigner, Largest, BIG4, ESG) = ROE \quad (1)$$

[The model that includes management strategy information to the standard model for forecasting firm management performance]

$$F\begin{pmatrix} SIZE, Leverage, Current, Growth, Foreigner, Largest, BIG4, ESG, Finance, \\ Customer, Internal, Learning \quad Growth, Sustainability \end{pmatrix}$$

$$= ROE \quad (2)$$

The following Fig 1 is a schematic diagram of how to compare and analyze the performance of the corporate management performance prediction model using the machine learning and deep learning classification method in this paper. This study uses eight machine learning and deep learning classifiers: K-Nearest Neighbors (KNN), Support Vector Machine (SVM), Gradient Boosting Machine (GBM), CatBoost, Generative Adversarial Networks (GAN), Recurrent Neural Networks (RNN), Long Short-Term Memory Networks (LSTM), Transformer.

Fig.1

Fig 1. Research model

Classification method

Cross-validation is a robust method for evaluating model performance, effectively mitigating the risks of overfitting and underfitting. This technique systematically partitions the dataset into multiple subsets, each sequentially employed for training and validation, thereby comprehensively assessing the model's generalization capabilities. Additionally, cross-validation facilitates the evaluation of the model across diverse data patterns instead of relying solely on a single train-test split. This approach establishes an essential baseline for the early identification of potential overfitting or underfitting and supports the optimization of the model. In this study, the K-fold cross-validation method, widely recognized for its reliability, was utilized with K set to 5. This method divided the dataset into five folds, each

serving as the validation set once, while the remaining folds were used for training.

This paper investigates classification techniques to develop machine learning and deep learning models for predicting firm management performance. In machine and deep learning, classification involves assigning input data to predefined categories or classes [27, 28]. Typically, models employ softmax functions in the final output layer to produce probability scores for each class, with the highest probability being selected for the prediction [29].

Machine learning and deep learning models often rely on a cross-entropy loss function to solve classification tasks, quantifying the difference between the predicted class probabilities and the actual class labels [30]. The model parameters are adjusted to minimize this difference, enhancing the model's accuracy [31]. The performance of these models is evaluated using key metrics such as accuracy, precision, recall, AUC (Area Under the ROC Curve), and F1 score [32].

Accuracy indicates the frequency with which the model's predictions align with the actual class labels, serving as a general measure of the model's effective-ness [33]. Precision refers to the ratio of true positive predictions to all positive predictions, thereby assessing the model's accuracy in identifying positive instances [34]. Recall measures the proportion of true positive cases correctly identified by the model, indicating its ability to capture all relevant instances [35]. AUC evaluates binary classification models by assessing their ability to distinguish between positive and negative classes through the ROC (Receiver Operating Characteristic) curve [36]. This curve plots the True Positive Rate (TPR) against the False Positive Rate (FPR) at various classification thresholds [37]. AUC values range from 0 to 1, with higher values indicating better model performance [38]. The F1 score is the harmonic mean of precision and recall, providing a balanced assessment of the model's classification

performance, particularly in cases with imbalanced data [39]. The F1 score also ranges from 0 to 1, with higher values indicating superior classification ability [40].

This study highlights the significance of these metrics in evaluating the effectiveness of machine learning models for predicting management performance. It offers insights into selecting and optimizing classification techniques to enhance predictive accuracy.

Classifier

Cross-validation is a reliable method for assessing a model's performance while guarding against overfitting and underfitting [41]. This technique divides the dataset into multiple segments, each used alternately for training and validation. This process helps to obtain a robust evaluation of the model's ability to generalize to unseen data [42].

Furthermore, cross-validation enables the model to be tested on various data patterns rather than restricted to a single train-test split [43]. This approach is crucial for detecting overfitting or underfitting and helps fine-tune the model for better performance.

This study employed the widely used K-fold cross-validation method, with K set to 5. This approach splits the dataset into five folds, each used as a validation set once, and the remaining folds serve as the training set. This ensures that every part of the dataset is used for training and validation, comprehensively assessing the model's performance.

K-Nearest Neighbors (KNN) is a straightforward, non-parametric algorithm for classification and regression [44]. The fundamental principle is classifying a data point by considering the majority class among its k nearest neighbors within the feature space [45]. In regression tasks, it averages the values of these k neighbors [44]. While KNN is simple and intuitive, its

computational cost can become significant as the dataset size increases since it requires calculating distances to every other point [46].

Support Vector Machine (SVM) is a supervised learning algorithm primarily applied to classification problems [47]. It identifies the hyperplane that most effectively separates the data into distinct classes [48]. The optimal hyperplane maximizes the margin between the classes [47]. SVMs perform well in high-dimensional spaces, making them particularly effective when the number of dimensions exceeds the number of samples [49]. However, they may struggle with noisy data and are sensitive to the choice of kernel [48].

Gradient Boosting Machine (GBM) is an ensemble technique that constructs models sequentially, with each new model correcting the errors of its predecessor [50]. The "boosting" refers to combining models to enhance accuracy [51]. GBM is widely used for tasks involving structured data, such as classification, regression, and ranking [52]. Despite its ability to produce strong predictive models, GBM is prone to overfitting and requires careful tuning of hyperparameters [50, 51].

CatBoost is a gradient-boosting algorithm specifically tailored to handle categorical data [53]. It processes categorical features directly without converting them into numerical values, such as through one-hot encoding [54]. Compared to other boosting algorithms, CatBoost stands out for its high performance and reduced need for extensive hyperparameter tuning [53]. It is particularly favored in fields like finance and e-commerce, where categorical data is prevalent [55].

Generative Adversarial Network (GAN) involves two neural networks, a generator and a discriminator, that engage in a competitive learning process [56]. The generator produces synthetic data samples, while the discriminator distinguishes between real and synthetic data

[57]. This adversarial approach allows GANs to generate highly realistic data, including images and videos [58]. GANs are extensively used in image generation, style transfer, and data augmentation, though they can be challenging to train and require careful tuning of hyperparameters [56, 58].

Recurrent Neural Network (RNN) is designed to process sequential data [59]. Unlike feedforward networks, RNNs have cyclical connections that allow them to retain the memory of previous inputs, making them well-suited for tasks like time series prediction, natural language processing, and speech recognition [60]. However, RNNs can encounter issues such as vanishing and exploding gradients, which can complicate the training process [61].

Long-short-term memory (LSTM) networks are a specialized type of RNN engineered to handle long-term dependencies in sequence data [62]. LSTM uses gating mechanisms to manage the flow of information, allowing them to preserve important information over extended sequences and mitigate the vanishing gradient problem [63]. It is widely used in text generation, machine translation, and forecasting time series [64].

Transformer is a neural network architecture that leverages self-attention mechanisms to process sequential data [65]. Unlike RNN, the transformer does not rely on sequential data processing; instead, it enables the model to evaluate the entire sequence simultaneously by computing attention scores [66]. This approach makes transformers highly efficient and effective for language translation, text summarization, and speech recognition tasks [67]. Transformers form the basis of many cutting-edge models in natural language processing, such as BERT and GPT [65].

Each model has distinct advantages and applications, making it suitable for various machine learning tasks depending on the specific problem requirements. Given the diverse

characteristics, strengths, and limitations of these machine learning and deep learning classification algorithms, selecting and applying the appropriate algorithm based on the problem and dataset is crucial. This study uses eight machine learning classifiers to evaluate the performance of the firm's management performance prediction model, which incorporates management strategy information from business reports.

Research results

This study focuses on developing machine learning and deep learning models to predict corporate management performance. The models are constructed using financial ratios identified as significant in previous research on corporate management performance and strategies. The study conducts a comparative analysis between two predictive models: one that utilizes only financial ratios related to corporate management performance and strategy and another that enhances this model by incorporating management strategy information based on the SBSC (Sustainable Balanced Scorecard) framework. This comparison aims to assess the impact of including strategic management information on the predictive accuracy of the models.

Machine learning and deep learning classifiers used in this paper are KNN, SVM, GBM, CatBoost, GAN, RNN, LSTM, and Transformer. Since this study used machine learning and deep learning classification methods, the performance of these classifiers is evaluated using accuracy, precision, recall, AUC, and F1 score indicators.

Table 4 below presents the accuracy results for various models that predict corporate management performance. The KNN standard model initially achieved an accuracy of

0.7704, which increased to 0.7821 after incorporating management strategy information derived from the CEO messages in the sustainability management report. The SVM standard model had an accuracy of 0.7836, which improved to 0.7985 when management strategy information from the SBSC framework was added. The accuracy of the GBM standard model was 0.8007, which rose to 0.8141 with the inclusion of additional management strategy data. The accuracy of the CatBoost standard model was 0.7928, which improved to 0.8069 after incorporating management strategy information. The GAN model had a baseline accuracy of 0.8169, which increased to 0.8255 with the addition of management strategy data. The RNN model initially showed an accuracy of 0.8218, which improved to 0.8390 when management strategy information was included. The LSTM model had a standard accuracy of 0.8315, which rose to 0.8485 after integrating the SBSC framework's management strategy information. Finally, the Transformer model initially had an accuracy of 0.8467, which improved to 0.8467 by including management strategy data. These results highlight the positive impact of incorporating management strategy information on the predictive accuracy of various machine learning and deep learning models.

Table 4. Firm management performance prediction accuracy results

Model	Classifier	Accuracy	Classifier	Accuracy	Classifier	Accuracy	Classifier	Accuracy
**Standard model**	KNN	0.7704	SVM	0.7836	GBM	0.8007	CatBoost	0.7928
**Standard model + Management**		0.7821		0.7985		0.8141		0.8069

**strategy information**								
**Standard model**	GAN	0.8169	RNN	0.8218	LSTM	0.8315	Transformer	0.8467
**Standard model + Management strategy information**		0.8255		0.8390		0.8485		0.8467

Table 5 provides the precision scores for predicting corporate management performance across various models. The KNN standard model achieved a precision of 0.7623, which increased to 0.7717 after incorporating management strategy information from the CEO messages in the sustainability management report. The SVM standard model demonstrated a precision of 0.7734, which improved to 0.7870 by adding management strategy information based on the SBSC framework. The GBM standard model's precision in predicting corporate management performance was 0.7964. This increased to 0.8042 after integrating management strategy data. The CatBoost standard model had a precision score of 0.7891, which rose to 0.7854 when management strategy information was included. The GAN model recorded a precision of 0.8063 in predicting management performance, which improved to 0.8152 by adding management strategy information. The RNN standard model initially had a precision of 0.8114, which increased to 0.8287 when management strategy data was incorporated. The precision score for the LSTM standard model was 0.8274, which improved to 0.8390 after including management strategy information according to the SBSC framework. Finally, the Trans-former standard model achieved a precision of 0.8353, which increased to 0.8482 with

the inclusion of management strategy information. These results demonstrate that integrating management strategy information generally enhances the precision of various models in predicting corporate management performance.

Table 5. Firm management performance prediction precision results

Model	Classifier	Precision	Classifier	Precision	Classifier	Precision	Classifier	Precision
**Standard model**	KNN	0.7623	SVM	0.7734	GBM	0.7964	CatBoost	0.7891
**Standard model + Management strategy information**	KNN	0.7717	SVM	0.7870	GBM	0.8042	CatBoost	0.7854
**Standard model**	GAN	0.8063	RNN	0.8114	LSTM	0.8274	Transformer	0.8353
**Standard model + Management strategy information**	GAN	0.8152	RNN	0.8287	LSTM	0.8390	Transformer	0.8482

Table 6 displays various models' recall values for predicting corporate management performance. The KNN standard model achieved a recall of 0.7792, which improved to 0.7884 by including management strategy information extracted from the CEO messages in the sustainability management report. The SVM standard model had a recall of 0.7862, which

increased to 0.7990 when management strategy information based on the SBSC framework was incorporated. The recall for the GBM standard model was 0.7999, which was enhanced to 0.8123 by adding management strategy data. The CatBoost standard model initially showed a recall of 0.7910, which increased to 0.8087 upon incorporating management strategy information. The GAN model had a baseline recall of 0.8132, which improved to 0.8269 after adding management strategy information. The RNN model started with a recall of 0.8224, which rose to 0.8387 with the inclusion of management strategy information. The recall for the LSTM model was 0.8369, which improved to 0.8474 when management strategy information from the SBSC framework was included. Lastly, the Transformer model achieved a recall of 0.8486, which improved to 0.8575 by incorporating management strategy information. These results indicate that adding management strategy information generally enhances recall across different models, improving their ability to correctly identify positive corporate management performance.

Table 6. Firm management performance prediction recall results

**Model**	**Classifier**	**Recall**	**Classifier**	**Recall**	**Classifier**	**Recall**	**Classifier**	**Recall**
**Standard model**	KNN	0.7792	SVM	0.7862	GBM	0.7999	CatBoost	0.7910
**Standard model + Management strategy information**		0.7884		0.7990		0.8123		0.8087
**Standard model**	GAN	0.8132	RNN	0.8224	LSTM	0.8369	Transformer	0.8486

**Standard model + Management strategy information**	0.8269	0.8387	0.8474	0.8575

Table 7 provides the AUC results for predicting corporate management performance across various models. The KNN standard model achieved an AUC of 0.7685, which improved to 0.7748 when management strategy information from the CEO messages in the sustainability management report was included. The SVM standard model demonstrated an AUC of 0.7762, which increased to 0.7849 with the addition of management strategy information based on the SBSC framework. For the GBM standard model, the AUC in predicting corporate management performance was 0.7924. This increased to 0.8042 after incorporating management strategy information. The CatBoost standard model had an AUC of 0.7865, which improved to 0.7941 by including management strategy data. The GAN model recorded an AUC of 0.8017, which increased to 0.8164 when adding management strategy information. The RNN standard model had an AUC of 0.8192, which improved to 0.8254 by including management strategy data. For the LSTM standard model, the AUC was 0.8280, which increased to 0.8341 when management strategy information from the SBSC framework was incorporated. Finally, the Transformer standard model achieved an AUC of 0.8362, which improved to 0.8481 by adding management strategy information. These results show that integrating management strategy information generally enhances the AUC, improving the models' ability to predict corporate management performance across different algorithms.

Table 7. Firm management performance prediction AUC results

Model	Classifier	AUC	Classifier	AUC	Classifier	AUC	Classifier	AUC
**Standard model**	KNN	0.7685	SVM	0.7762	GBM	0.7924	CatBoost	0.7865
**Standard model + Management strategy information**		0.7748		0.7849		0.8042		0.7941
**Standard model**	GAN	0.8017	RNN	0.8192	LSTM	0.8280	Transformer	0.8362
**Standard model + Management strategy information**		0.8164		0.8254		0.8341		0.8481

Table 8 [illegible]ws the F1 scores for predicting corporate management performance across different models. The KNN standard model initially achieved an F1 score of 0.7731, which increased to 0.7848 after incorporating management strategy information from the CEO messages in the sustainability management report. The SVM standard model had an F1 score of 0.7860, which improved to 0.7997 with the inclusion of management strategy information based on the SBSC framework. For the GBM standard model, the F1 score was 0.8021, which increased to 0.8134 after adding management strategy data. The CatBoost standard model recorded an F1 score of 0.7932, which rose to 0.8044 with the addition of management strategy information. The GAN model initially had an F1 score of 0.8170, which improved to 0.8266 when management strategy data was incorporated. The RNN model had a standard F1

score of 0.8225, which increased to 0.8394 with the inclusion of management strategy information. For the LSTM model, the F1 score was 0.8372, which improved to 0.8495 after adding information on the SBSC framework management strategy. Finally, the Trans-former model achieved an F1 score of 0.8487, which increased to 0.8572 when management strategy information was incorporated. These results indicate that including management strategy information generally enhances the F1 score across various models, improving their overall performance in predicting corporate management outcomes.

Table 8. Firm management performance prediction F1 score results

Model	Classifier	F1 score	Classifier	F1 score	Classifier	F1 score	Classifier	F1 score
**Standard model**	KNN	0.7731	SVM	0.7860	GBM	0.8021	CatBoost	0.7932
**Standard model + Management strategy information**		0.7848		0.7997		0.8134		0.8044
**Standard model**	GAN	0.8170	RNN	0.8225	LSTM	0.8372	Transformer	0.8487
**Standard model + Management**		0.8266		0.8394		0.8495		0.8572

**strategy information**

Conclusion

This study applied machine learning and deep learning classification techniques, leveraging AI methods, to predict corporate management performance. The classifiers used included KNN, SVM, GBM, CatBoost, GAN, RNN, LSTM, and Transformer. By drawing on previous research, the study developed a predictive model using financial variables strongly linked to corporate management performance.

The research particularly focused on comparing two predictive models. One relied solely on financial variables relevant to firm management performance, and another incorporated management strategy information based on the SBSC framework alongside these financial variables. In this study, keywords were extracted after collecting the contents of the CEO message of the sustainability management report disclosed by the company by text mining techniques. The SBSC framework classified these keywords according to the financial perspective, customer perspective, internal process perspective, learning and growth perspective, and sustainability perspective and then included them in the prediction model.

The key findings from this study are as follows. First, the predictive model that included management strategy information alongside financial variables outperformed the model that relied solely on financial data. Second, among the eight machine learning and deep learning classifiers, the Transformer model delivered the highest accuracy in forecasting firm management performance, followed by LSTM, RNN, GAN, GBM, CatBoost, SVM, and

KNN. This suggests that Transformers are particularly well-suited for analyzing large-scale, time-series data like corporate financial information. Third, LSTM and RNN also proved effective, indicating that deep learning models may be better equipped for corporate financial data analysis than traditional machine learning models.

This paper's contributions are as follows. Academically, the study identified a more accurate method for predicting corporate management performance by incorporating qualitative management strategy data rather than relying solely on quantitative financial data. The study introduced an innovative approach by extracting management strategy information from CEO messages in sustainability reports using text mining and integrating this information into the predictive model. This methodology can potentially guide future research in predictive and convergent fields. The paper empirically compares machine learning and deep learning algorithms for predicting corporate management performance, offering valuable insights for researchers selecting algorithms for financial data analysis. The research establishes an academic foundation for integrating the SBSC framework into machine learning models, thereby contributing to the development of structured management strategy analysis.

Practically, this study offers several insights. Including non-financial corporate strategy information in the predictive model led to higher accuracy, providing actionable insights for more precise management performance predictions and aiding strategic decision-making for companies. The findings suggest that CEO messages in sustainability reports contain valuable information about a company's management strategy, offering external stakeholders, such as investors and creditors, a way to infer these strategies. The study's comparison of machine learning and deep learning models can help practitioners select the most effective model for predicting management performance using corporate financial data, with a particular

recommendation for using Transformer, LSTM, and RNN models.

In conclusion, this paper presents a creative and innovative research methodology that combines machine learning and deep learning with the SBSC framework. It proposes a new theoretical framework for predictive research using these advanced techniques while providing practical tools for stakeholders to make more informed decisions.

References

[1] Chen S, Song Y, Gao P. Environmental, social, and governance (ESG) performance and financial outcomes: Analyzing the impact of ESG on financial performance. J Environ Manag. 2023; 345:118829.2. Weston, P., & Nnadi, M. (2023). Evaluation of strategic and financial variables of corporate sustainability and ESG policies on corporate finance performance. Journal of Sustainable Finance & Investment, 13(2), 1058-1074.

[2] Weston P, Nnadi M. Evaluation of strategic and financial variables of corporate sustainability and ESG policies on corporate finance performance. J Sustain Financ Invest. 2023;13(2):1058-74.

[3] Na HJ, Lee KC, Choi SU, Kim ST. Exploring CEO messages in sustainability management re-ports: Applying sentiment mining and sustainability balanced scorecard methods. Sustainability. 2020;12(2):590.

[4] Stacchezzini R, Melloni G, Lai A. Sustainability management and reporting: the role of integrated reporting for communicating corporate sustainability management. J Clean Prod. 2016;136:102-10.

[5] Ashford SJ, Wellman N, Sully de Luque M, De Stobbeleir KE, Wollan M. Two

roads to effectiveness: CEO feedback seeking, vision articulation, and firm performance. J Organ Behav. 2018;39(1):82-95.

[6] Choi YG, Cho KT. Analysis of safety management characteristics using network analysis of CEO messages in the construction industry. Sustainability. 2020;12(14):5771.

[7] Lee J, Jang D, Park S. Deep learning-based corporate performance prediction model considering technical capability. Sustainability. 2017;9(6):899.

[8] Tanaka M, Bloom N, David JM, Koga M. Firm performance and macro forecast accuracy. J Monet Econ. 2020;114:26-41.

[9] Fuertes G, Alfaro M, Vargas M, Gutierrez S, Ternero R, Sabattin J. Conceptual framework for the strategic management: a literature review—descriptive. J Eng. 2020;2020(1):6253013.

[10] Iyer VH, Gupta U, Somani PP, Behare N, Mahajan R, Singh M. The Rise and Rise of Artificial Intelligence in the World of Finance. In: Risks and Challenges of AI-Driven Finance: Bias, Ethics, and Security. IGI Global; 2024. p. 35-56.

[11] Kang Y, Cai Z, Tan CW, Huang Q, Liu H. Natural language processing (NLP) in management research: A literature review. J Manag Anal. 2020;7(2):139-72.

[12] Sarica S, Luo J. Stopwords in technical language processing. PLoS One. 2021;16(8).

[13] Mielke SJ, Alyafeai Z, Salesky E, Raffel C, Dey M, Gallé M, et al. Between words and char-acters: A brief history of open-vocabulary modeling and tokenization in NLP. arXiv preprint arXiv:2112.10508. 2021.

[14] HaCohen-Kerner Y, Miller D, Yigal Y. The influence of preprocessing on text classification using a bag-of-words representation. PLoS One. 2020;15(5).

[15] Addiga A, Bagui S. Sentiment analysis on Twitter data using term frequency-inverse

document frequency. J Comput Commun. 2022;10(8):117-28.

[16] Zandieh M, Shariat SY, Rabieh M, Tootooni M. A new framework for dynamic sustainability balanced scorecard in order to strategic decision making in a turbulent environment. J Ind Syst Eng. 2020;12(4):107-35.

[17] Tuori MA, Rois RSR, Martowidjojo YAN, Pasang H. Formulating a sustainability balanced scorecard (SBSC) for a leading integrated palm oil based consumer products public company in Indonesia. J Manag Inf Decis Sci. 2021;24(3):1-15.

[18] Kijkasiwat P, Phuensane P. Innovation and firm performance: The moderating and mediating roles of firm size and small and medium enterprise finance. J Risk Financ Manag. 2020;13(5):97.

[19] Doan T. Financing decision and firm performance: Evidence from an emerging country. Manag Sci Lett. 2020;10(4):849-54.

[20] Effiong SA, Ejabu FE. Liquidity risk management and financial performance: are consumer goods companies involved. Int J Recent Technol Eng. 2020;9(1):580-9.

[21] Putri IGAPT, Rahyuda H. Effect of capital structure and sales growth on firm value with prof-itability as mediation. Int Res J Manag IT Soc Sci. 2020;7(1):145-55.

[22] Tsafack G, Guo L. Foreign shareholding, corporate governance and firm performance: Evidence from Chinese companies. J Behav Exp Financ. 2021;31:100516.

[23] Gupta K, Crilly D, Greckhamer T. Stakeholder engagement strategies, national institutions, and firm performance: A configurational perspective. Strateg Manag J. 2020;41(10):1869-900.

[24] Dawood M, ur Rehman S, Majeed U, Idress S. Contribution the Effect of Corporate Governance on Firm Performance in Pakistan. Rev Educ Adm Law. 2023;6(1):51-

62.
[25] Mohapatra S, Pattanayak JK. Unraveling the Dynamics of Intellectual Capital, Firm Perfor-mance, and the Influential Moderators—BIG4 Auditors and Group Affiliation. Int J Financ Stud. 2024;12(1):29.
[26] Huang DZ. Environmental, social and governance (ESG) activity and firm performance: A re-view and consolidation. Account Financ. 2021;61(1):335-60.
[27] Mehbodniya A, Alam I, Pande S, Neware R, Rane KP, Shabaz M, et al. [Retracted] Financial Fraud Detection in Healthcare Using Machine Learning and Deep Learning Techniques. Secur Commun Netw. 2021;2021(1):9293877.
[28] Singh V, Chen SS, Singhania M, Nanavati B, Gupta A. How are reinforcement learning and deep learning algorithms used for big data based decision making in financial industries–A re-view and research agenda. Int J Inf Manag Data Insights. 2022;2(2):100094.
[29] Maharjan S, Alsadoon A, Prasad PWC, Al-Dalain T, Alsadoon OH. A novel enhanced softmax loss function for brain tumour detection using deep learning. J Neurosci Methods. 2020;330:108520.
[30] Li L, Doroslovački M, Loew MH. Approximating the gradient of cross-entropy loss function. IEEE Access. 2020;8:111626-35.
[31] Nguyen H, Vu T, Vo TP, Thai HT. Efficient machine learning models for prediction of concrete strengths. Constr Build Mater. 2021;266:120950.
[32] Nabil A, Seyam M, Abou Elfetouh A. Prediction of students' academic performance based on courses' grades using deep neural networks. IEEE Access. 2021;9:140731-46.
[33] Kuo KM, Talley PC, Chang CS. The accuracy of machine learning approaches using

non-image data for the prediction of COVID-19: A meta-analysis. Int J Med Inform. 2022;164:104791.

[34] Luan H, Tsai CC. A review of using machine learning approaches for precision education. Educ Technol Soc. 2021;24(1):250-66.

[35] Gupta A, Anand A, Hasija Y. Recall-based Machine Learning approach for early detection of Cervical Cancer. In: 2021 6th International Conference for Convergence in Technology (I2CT). IEEE; 2021. p. 1-5.

[36] Carrington AM, Fieguth PW, Qazi H, Holzinger A, Chen HH, Mayr F, et al. A new concord-ant partial AUC and partial c statistic for imbalanced data in the evaluation of machine learning algorithms. BMC Med Inform Decis Mak. 2020;20:1-12.

[37] Yao Y, Lin Q, Yang T. Large-scale optimization of partial auc in a range of false positive rates. Adv Neural Inf Process Syst. 2022;35:31239-53.

[38] Carrington AM, Fieguth PW, Qazi H, Holzinger A, Chen HH, Mayr F, et al. A new concord-ant partial AUC and partial c statistic for imbalanced data in the evaluation of machine learning algorithms. BMC Med Inform Decis Mak. 2020;20:1-12.

[39] Chicco D, Jurman G. The advantages of the Matthews correlation coefficient (MCC) over F1 score and accuracy in binary classification evaluation. BMC Genomics. 2020;21:1-13.

[40] Yacouby R, Axman D. Probabilistic extension of precision, recall, and f1 score for more thorough evaluation of classification models. In: Proceedings of the first workshop on evaluation and comparison of NLP systems. 2020. p. 79-91.

[41] Tougui I, Jilbab A, El Mhamdi J. Impact of the choice of cross-validation techniques on the results of machine learning-based diagnostic applications. Healthc Inform Res. 2021;27(3):189-99.

[42] Nti IK, Nyarko-Boateng O, Aning J. Performance of machine learning algorithms with differ-ent K values in K-fold CrossValidation. Int J Inf Technol Comput Sci. 2021;13(6):61-71.

[43] Scheda R, Diciotti S. Explanations of machine learning models in repeated nested cross-validation: an application in age prediction using brain complexity features. Appl Sci. 2022;12(13):6681.

[44] Xiong L, Yao Y. Study on an adaptive thermal comfort model with K-nearest-neighbors (KNN) algorithm. Build Environ. 2021;202:108026.

[45] Boateng EY, Otoo J, Abaye DA. Basic tenets of classification algorithms K-nearest-neighbor, support vector machine, random forest and neural network: A review. J Data Anal Inf Process. 2020;8(4):341-57.

[46] Bansal M, Goyal A, Choudhary A. A comparative analysis of K-nearest neighbor, genetic, support vector machine, decision tree, and long short term memory algorithms in machine learning. Decis Anal J. 2022;3:100071.

[47] Kurani A, Doshi P, Vakharia A, Shah M. A comprehensive comparative study of artificial neural network (ANN) and support vector machines (SVM) on stock forecasting. Ann Data Sci. 2023;10(1):183-208.

[48] Yu Y, Shao M, Jiang L, Ke Y, Wei D, Zhang D, et al. Quantitative analysis of multiple com-ponents based on support vector machine (SVM). Optik. 2021;237:166759.

[49] Roy A, Chakraborty S. Support vector machine in structural reliability analysis: A review. Re-liab Eng Syst Saf. 2023;233:109126.

[50] Konstantinov AV, Utkin LV. Interpretable machine learning with an ensemble of gradient boosting machines. Knowl-Based Syst. 2021;222:106993.

[51] Deif MA, Hammam RE, Solyman AA. Gradient boosting machine based on PSO for prediction of leukemia after a breast cancer diagnosis. Int J Adv Sci Eng Inf Technol. 2021;11(2):508-15.

[52] Nabi RM, Soran Ab M S, Harron H. A novel approach for stock price prediction using gradient boosting machine with feature engineering (gbm-wfe). Kurdistan J Appl Res. 2020;5(1):28-48.

[53] Ibrahim AA, Ridwan RL, Muhammed MM, Abdulaziz RO, Saheed GA. Comparison of the CatBoost classifier with other machine learning methods. Int J Adv Comput Sci Appl. 2020;11(11).

[54] Luo M, Wang Y, Xie Y, Zhou L, Qiao J, Qiu S, et al. Combination of feature selection and catboost for prediction: The first application to the estimation of aboveground biomass. Forests. 2021;12(2):216.

[55] Nguyen N, Duong T, Chau T, Nguyen VH, Trinh T, Tran D, et al. A proposed model for card fraud detection based on Catboost and deep neural network. IEEE Access. 2022;10:96852-61.

[56] Durgadevi M. Generative Adversarial Network (GAN): A general review on different variants of GAN and applications. In: 2021 6th International Conference on Communication and Electronics Systems (ICCES); 2021. p. 1-8.

[57] Vaccari I, Orani V, Paglialonga A, Cambiaso E, Mongelli M. A generative adversarial network (GAN) technique for internet of medical things data. Sensors. 2021;21(11):3726.

[58] Lim W, Chek KYS, Theng LB, Lin CTC. Future of generative adversarial networks (GAN) for anomaly detection in network security: A review. Comput Secur. 2024;103733.

[59] Sherstinsky A. Fundamentals of recurrent neural network (RNN) and long short-term memory (LSTM) network. Physica D. 2020;404:132306.
[60] Bouarara HA. Recurrent neural network (RNN) to analyse mental behaviour in social media. Int J Softw Sci Comput Intell. 2021;13(3):1-11.
[61] Dhruv P, Naskar S. Image classification using convolutional neural network (CNN) and recur-rent neural network (RNN): A review. In: Machine learning and information processing: proceedings of ICMLIP 2019. Springer; 2020. p. 367-81.
[62] Laghrissi F, Douzi S, Douzi K, Hssina B. Intrusion detection systems using long short-term memory (LSTM). J Big Data. 2021;8(1):65.
[63] Huang R, Wei C, Wang B, Yang J, Xu X, Wu S, et al. Well performance prediction based on Long Short-Term Memory (LSTM) neural network. J Pet Sci Eng. 2022;208:109686.
[64] Lees T, Reece S, Kratzert F, Klotz D, Gauch M, De Bruijn J, et al. Hydrological concept formation inside long short-term memory (LSTM) networks. Hydrol Earth Syst Sci Discuss. 2021;2021:1-37.
[65] Xie J, Zhang J, Sun J, Ma Z, Qin L, Li G, et al. A transformer-based approach combining deep learning network and spatial-temporal information for raw EEG classification. IEEE Trans Neural Syst Rehabil Eng. 2022;30:2126-36.
[66] Li Y, Cao J, Xu Y, Zhu L, Dong ZY. Deep learning based on Transformer architecture for power system short-term voltage stability assessment with class imbalance. Renew Sustain Energy Rev. 2024;189:113913.
[67] Borhani Y, Khoramdel J, Najafi E. A deep learning based approach for automated plant dis-ease classification using vision transformer. Sci Rep. 2022;12(1):11554.

Figure

Click here to access/download;Figure;fig 1.tiff

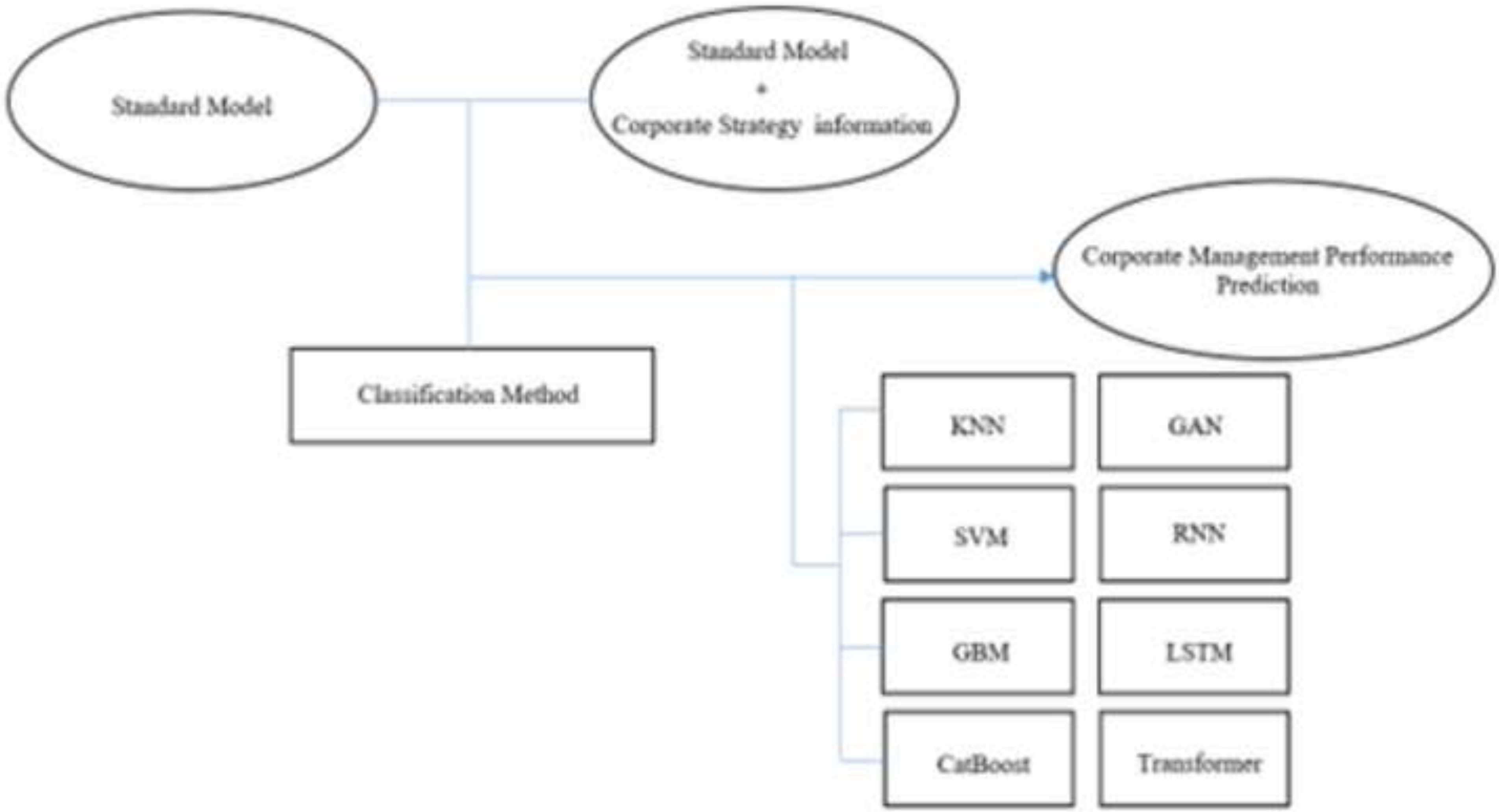

Supplement: Attachment — Submitted filename: PONE-D-24-38231_reviewerForAuthors.pdf [file pone.0347140.s002.pdf]
