# Supplementary material for: Predicting corporate management performance using AI: Incorporating CEO strategy insights from sustainable management reports
Source: PLoS One. 2026 May 6;21(5):e0347140. doi: 10.1371/journal.pone.0347140 (PMC13148710; doi:10.1371/journal.pone.0347140)
Supplement: Attachment — Submitted filename: my_comments_to_authors.docx [file pone.0347140.s001.docx]

Dear Authors, I enjoyed reading your manuscript ,

Your study shows a clear and consistent improvement in the performance of machine learning and deep learning models when management strategy information (derived from the CEO’s messages in sustainability reports) is incorporated alongside financial data. This finding is significant as it highlights the added value of non-financial information in predicting corporate management performance. Furthermore, the results of all the evaluation metrics are consistent (accuracy, precision, recall, AUC, and F1 score), showing that integrating management strategy data leads to improvements in the predictive performance of most models. The results for deep learning models, particularly the Transformer, LSTM, and RNN, are quite interesting, they outperform traditional machine learning models, highlighting their suitability for handling complex, time-series data in corporate financial analysis. Plus, the use of the SBSC framework to incorporate management strategy information is quite innovative and novel, demonstrating the integration of qualitative data into quantitative predictive models.

I have just a few recommendations, most of them are how the sentences should be re-structured, the science itself is sound.

The abbreviation ESG in the introduction should be explained in its full-form first -environmental, social and governance (ESG)

Content beginning at line 74 is slightly ambiguous:

*My suggestion for improvement of content:*

Should text mining techniques be applied to refine the company's financial data and the management strategy information from the CEO's message in the non-financial sustainability report, the model's predictive performance would be expected to improve significantly.

Content between at line 77-84 is ambiguous:

*My suggestion for improvement of content:*

In summary, the aim of this study was to develop an AI-based predictive model which would predict the performance management of a company with accuracy by analysing the information normally contained within the CEO’s report on sustainability-management. The model predicting the performance of management using analysis of strategic information by management that typically comprises financial data from the corporate sector with environmental, social and governance (ESG) activities would be expected to perform better than any existing prediction models on the performance of management. The prediction model discussed in this paper was previously validated in studies in the field of accounting using strategically selected financial variables.

This study's findings are expected to inspire new academic research on predictive models for evaluating management performance and to harmonise academic-research fields between that of business administration and that of computer engineering. The results of this study is also expected to aid the decision-making process of various stakeholders in a company.

**Content between 90-106 is ambiguous, my suggestion for improvement is as follows:.**

This study distinguishes itself from previous research in several significant ways. Unlike earlier studies that relied on a broad range of financial data with limited connection to accounting theory, this study develops a model grounded in accounting research, focusing on financial variables directly related to management performance. A key innovation is the incorporation of CEO messages extracted from sustainability management reports, which were processed using text mining techniques. By integrating these non-financial insights with financial data, the predictive performance of the model was substantially improved.

Furthermore, this study employs both machine learning and deep learning techniques across eight different models, ensuring a customer-oriented, highly reliable predictive approach. By comparing models that use only financial information with those that include strategic, non-financial data, the research clearly shows that adding strategic insights enhances predictive accuracy. This study contributes academically by highlighting the role of ESG activities in management strategies and provides actionable information for promoting sustainable corporate growth.

**Methods section of DATA between 110 – 151 could be better structured: my suggestion:**

The publicly available sustainability management report provides essential details about a company’s ESG activities, vision, and strategic objectives [4]. Of particular interest is the CEO’s message, which subtly reveals the company’s strategic direction [3].

In this study, text data from sustainability management reports was extracted using text mining techniques and processed with Natural Language Processing (NLP) methods [11]. During preprocessing, text normalization was applied to remove unnecessary punctuation and special characters. Stop Word Removal filtered out irrelevant or redundant terms [12]. Next, tokenization segmented the text into meaningful units such as words or sentences [13]. The processed text was then converted into vector representations using the Bag of Words (BoW) model [14]. Significant keywords were identified by calculating their TF-IDF (Term Frequency-Inverse Document Frequency) values, with higher values indicating greater importance [15].

Keywords with a TF-IDF value of 1.5 or higher were selected and categorized according to the SBSC framework. This framework classifies keywords into five perspectives: financial, customer, internal process, learning and growth, and sustainability [16, 17]. The classification criteria emphasized the dominant perspective of the extracted keywords from each CEO’s message. For instance, if five keywords aligned with the finance perspective, three with the customer perspective, and two with internal processes, the company’s strategy was deemed to prioritize finance. In such cases, a value of 1 was assigned to the finance variable, while 0 was assigned to the remaining SBSC categories. Through this method, the study quantified the management strategy emphasized by each company.

The management strategy data was sourced from sustainability management reports available on the ESG Portal Site (<https://esg.krx.co.kr>). The CEO’s message within these reports summarizes the company’s annual vision, goals, and business outlook. Financial data was obtained from NICE Credit Rating Information’s Value Search platform (<http://www.nicerating.com>). The analysis covered the period from 2016 to 2023 and focused on companies listed on KOSPI and KOSDAQ.

Initially, the dataset comprised 16,343 companies listed on KOSPI and KOSDAQ between 2016 and 2023, excluding those in the financial sector. Several exclusions were applied: 385 companies that did not close their accounts in December, 993 with unavailable financial data, 35 with capital erosion due to sustained losses, and 8,455 that lacked ESG ratings from the Korea ESG Standards Institute. Additionally, 5,604 companies that did not disclose a sustainability management report, including a CEO message, were excluded, as this information was essential for the prediction model.

**Some detail needed for the methods:**

What type of toolkits were used for NLP? Was it NLTK , SpaCy, Stanford CoreNLP, Hugging Face transformers, OpenNLP or AllenNLP? Kindly elaborate.

**Include a sentence, something to the effect of:**

Bag of Words (BoW) converts text into numerical vectors by mapping word frequencies or presence in a document based on a vocabulary of unique words, ignoring order and grammar.

What library was used for BoW implementation? Was it using Scikit-learn’s Countvectorizer or TfidVectorizer? Or did the authors use NLTK?

**The rationale for exclusion of companies in Table-1 could be explained better, this would enhance the reader’s understanding of the study design perhaps in a figure or separate table as follows:**

1. Companies in the financial industry – ie they have different structures, regulatory requirements, reporting standards—and excluding them ensures a more homogenous dataset and prevents sector-specific factors from skewing the results
2. Companies not closing accounts in December: - companies with non-standard fiscal years could complicate data consistency – this would enhance uniformity in financial data analysis.
3. Companies without available financial data (993).

Financial data is critical for input to the model of this study

4. Companies with capital erosion due to sustained losses

Such companies might represent extreme cases or outliers – potentially distorting the analysis. This exclusion ensures the focus is on industries with standard operating conditions.

5. Companies without ESG ratings

ESG ratings are crucial to this study as they provide key non-financial info for the predictive model.

6. Companies without CEO messages in sustainability reports:

As this study relies strongly on CEO messages for management strategy insights, those without this information cannot be analysed under the study’s framework.

**The content between lines 157 and 170 could be better structured perhaps using bullets, my suggestion would be as follows:**

The **Finance**, **Customer**, **Internal Process**, **Learning & Growth**, and **Sustainability** variables are all dummy variables. These variables are assigned a value of 1 if the most frequently repeated keywords in the CEO’s message of the **Sustainable Management Report** are related to the respective categories; otherwise, the value is 0. Specifically:

- **Finance** is 1 if the dominant keywords are related to finance, and 0 otherwise.
- **Customer** is 1 if the dominant keywords relate to customers, and 0 otherwise.
- **Internal Process** is 1 if the dominant keywords pertain to internal processes, and 0 otherwise.
- **Learning & Growth** is 1 if the dominant keywords are linked to employee education and growth, and 0 otherwise.
- **Sustainability** is 1 if the most frequent keywords are related to environmental issues, donations, or sustainable growth, and 0 otherwise.
