# Supplementary material for: Predicting corporate management performance using AI: Incorporating CEO strategy insights from sustainable management reports
Source: PLoS One. 2026 May 6;21(5):e0347140. doi: 10.1371/journal.pone.0347140 (PMC13148710; doi:10.1371/journal.pone.0347140)
Supplement: Attachment — Submitted filename: Response to Reviewer 2_PONE-D-24-38231R1.docx [file pone.0347140.s003.docx]

**Response to Reviewer #2**

PONE-D-24-38231R1

Predicting Corporate Management Performance Using AI: Incorporating CEO Strategy Insights from Sustainable Management Reports

*We are grateful for the time and effort devoted to reviewing our manuscript. The comments and suggestions were highly valuable and have guided revisions that improve the manuscript’s clarity, robustness, and presentation. Below we provide a detailed, point-by-point response describing the revisions made.*

Reviewer #2:

I hope and look forward to seeing future work from your group on this topic which would explore alternative data sources and perhaps also test models across different industries.

[Response]

Thank you for this constructive suggestion. We have strengthened the Discussion by adding an explicit “future research” extension paragraph that outlines two concrete directions. First, we propose expanding beyond CEO messages in sustainability reports by integrating alternative text- and event-based data sources such as earnings-call transcripts, MD&A/annual reports, investor presentations, analyst reports, ESG assurance statements, and firm-level news/market-event signals) to mitigate source-specific biases and improve robustness. Second, we propose cross-industry validation by stratifying the sample by industry and testing both industry heterogeneity in the strategy–performance mapping and cross-industry transferability of the trained models. All added text is highlighted in yellow in the revised manuscript.

[Modification]

In closing, this study not only improves the precision of corporate performance prediction but also introduces a novel methodology that combines AI, ESG, and strategic discourse. Future research may extend this work by testing its generalizability across different industries and geographies, incorporating sentiment or topic modeling, or examining the influence of CEO characteristics on message tone and structure. Overall, this research offers a robust and replicable framework for next-generation performance analytics, responsive to both the data realities and strategic complexities of modern corporate governance. Future research will extend this framework by integrating alternative data sources and by validating cross-industry generalizability through sector-stratified experiments.

*We believe these revisions have strengthened the manuscript substantially. Thank you again for your thoughtful evaluation and guidance. We would be pleased to provide any additional clarifications if needed.*
