# Supplementary material for: Predicting corporate management performance using AI: Incorporating CEO strategy insights from sustainable management reports
Source: PLoS One. 2026 May 6;21(5):e0347140. doi: 10.1371/journal.pone.0347140 (PMC13148710; doi:10.1371/journal.pone.0347140)
Supplement: Attachment — Submitted filename: Response to Reviewer 1.docx [file pone.0347140.s004.docx]

**Response to Reviewer #1**

PONE-D-24-38231R1

Predicting Corporate Management Performance Using AI: Incorporating CEO Strategy Insights from Sustainable Management Reports

*We are grateful for the time and effort devoted to reviewing our manuscript. The comments and suggestions were highly valuable and have guided revisions that improve the manuscript’s clarity, robustness, and presentation. Below we provide a detailed, point-by-point response describing the revisions made.*

Reviewer #1:

-You should consider adding a brief summary of key performance results to the abstract so readers can quickly grasp the main contributions and outcomes of the study.

[Response]

We revised the Abstract to include a concise summary of key performance results so readers can quickly grasp the main outcomes. Specifically, we added one sentence reporting the best-performing model’s Accuracy/AUC/F1 under 5-fold cross-validation and the average performance gains achieved by incorporating SBSC-based strategy indicators. The newly added text is highlighted in yellow in the revised manuscript.

[Modification]

# **Abstract**

This study proposes an AI-based model to predict corporate management performance by combining financial data with strategic information extracted from CEO messages in sustainability reports. Using a dataset of 1,271 listed companies on Korea’s KOSPI and KOSDAQ markets (2016–2023), we applied eight machine learning and deep learning classifiers: KNN, SVM, GBM, CatBoost, GAN, RNN, LSTM, and Transformer. Financial variables were selected based on prior accounting research, while strategic variables were derived via text mining of CEO messages and categorized using the Sustainable Balanced Scorecard (SBSC) framework. Results show that models incorporating both financial and strategy-based variables outperformed those using financial data alone. Notably, the Transformer model achieved the highest predictive accuracy, followed by LSTM and RNN. These findings provide actionable insights for investors and corporate stakeholders while advancing interdisciplinary research between accounting and AI. Under 5-fold cross-validation, the best-performing hybrid model (Transformer with SBSC features) achieved Accuracy=0.8467, AUC=0.8481, and F1=0.8572, and adding SBSC strategy indicators improved mean performance across models (ΔAccuracy=+0.0121; ΔAUC=+0.0092; ΔF1=+0.0119).

- The introduction would benefit from a more explicit justification of the need for this study.

[Response]

We strengthened the Introduction by adding an explicit justification for the need for this study. Specifically, we inserted a new paragraph highlighting (1) the rapid expansion of ESG disclosure and stakeholder reliance on sustainability information, (2) the limited evidence on whether CEO narrative strategy signals provide incremental predictive value beyond conventional accounting ratios, and (3) the methodological need for a transparent, theory-grounded (SBSC-based) approach to convert CEO discourse into actionable features for performance prediction. The added text is highlighted in yellow in the revised manuscript.

[Modification]

# **Introduction**

In recent years, environmental, social, and governance (ESG) considerations have emerged as a central focus of global corporate management [1]. Consequently, firms are increasingly formulating their management strategies in alignment with ESG principles [2]. Amid these evolving managerial paradigms, developing accurate predictive models for corporate performance has become a pressing academic and practical challenge.

While traditional approaches to performance forecasting have relied heavily on financial indicators [7, 8], such data alone cannot fully capture a firm’s strategic orientation or sustainability posture. Corporate reports often contain non-financial disclosures that provide meaningful insights into strategic direction. In particular, sustainability management reports have gained prominence as critical sources of ESG-related information. Notably, the CEO’s message within these reports often articulates a company’s long-term vision, goals, and strategic intent in a concise and representative manner [3, 4, 5, 6].

However, despite the rapid expansion of ESG disclosure and the growing reliance of investors and regulators on sustainability information, there is still limited evidence on whether the strategic narratives embedded in sustainability reports can systematically improve firm-performance prediction beyond conventional accounting ratios [1,2,7,8]. Prior forecasting studies typically treat non-financial text as anecdotal or rely on less interpretable representations, leaving a methodological gap for a transparent, theory-grounded translation of CEO discourse into actionable strategic features. Addressing this gap is important because misclassifying future management performance can lead to inefficient capital allocation and delayed risk responses, especially in ESG-sensitive markets. Therefore, a hybrid and interpretable AI framework that integrates validated financial variables with SBSC-mapped CEO strategy signals is needed.

Unlike prior studies that utilize financial statements or MD&A sections to infer corporate strategies, this study leverages the CEO's message from sustainability management reports as the primary source for extracting strategic insights. This approach reflects the growing importance of ESG in corporate governance and strategy formulation. By applying text mining techniques to these messages and classifying the extracted content using the Sustainable Balanced Scorecard (SBSC) framework, this study integrates qualitative strategic orientation into a machine learning-based corporate performance prediction model.

Furthermore, this research addresses the limitations of prior models that neglected the role of strategic, non-financial data in predictive accuracy. By quantifying the management strategy embedded in ESG disclosures, this study not only augments existing financial models but also introduces a hybrid approach that aligns with emerging sustainability paradigms. The proposed model is expected to offer enhanced predictive validity and provide valuable decision-making insights for corporate managers, investors, and stakeholders concerned with long-term performance and sustainable growth [9].

Predictive technology has advanced significantly due to the rapid development of artificial intelligence-based machine learning and deep learning technologies [10]. These technologies are particularly effective in analyzing diverse and complex datasets. In this context, should text mining techniques be applied to refine the company’s financial data and the management strategy information from the CEO’s message in the non-financial sustainability report, the model's predictive performance would be expected to improve significantly. Incorporating such refined information into the predictive model may further enhance its accuracy and reliability, as recent studies suggest that integrating structured and unstructured data contributes meaningfully to performance improvements.

In other words, this paper aims to develop an AI-based prediction model that can more accurately predict a company's management performance by reflecting the information contained in the CEO message of the sustainability management report. The management performance prediction model that reflects management strategy information, including corporate financial data and ESG activities, is expected to perform better than existing models. This model is systematically and logically designed using financial variables that have already been validated in prior accounting research.

In summary, the aim of this study was to develop an AI-based predictive model which would predict the performance management of a company with accuracy by analyzing the information normally contained within the CEO’s report on sustainability management. The proposed model, which integrates strategic management information—particularly financial data and ESG activities—is anticipated to outperform traditional models in forecasting managerial performance. Furthermore, this study’s findings are expected to inspire new academic research in predictive analytics and bridge interdisciplinary gaps between business administration and computer engineering. The results also offer practical value by supporting more informed decision-making for various corporate stakeholders.

This study differs from prior research in several meaningful ways, both methodologically and conceptually. First, while earlier studies often utilized broad financial datasets without a strong foundation in accounting theory, this study constructs a predictive model grounded in validated financial variables derived from accounting literature, focusing on those with a direct link to management performance.

Second, this research uniquely incorporates strategic, non-financial insights by extracting and analyzing CEO messages from sustainability management reports using text mining techniques—a novel approach not previously applied in this context. While CEO messages are often seen as symbolic or aspirational, this study demonstrates that when systematically processed, they contain strategic signals that can meaningfully contribute to performance prediction. This approach aligns with current trends emphasizing the role of ESG and sustainability discourse in corporate decision-making.

Third, by applying eight machine learning and deep learning classification models—including Transformer and LSTM architectures—the study provides a robust comparative framework. Results show that models incorporating CEO message data outperform those relying solely on financial variables, indicating that strategic narratives provide additional explanatory power.

From a practical standpoint, the methodology can be applied in investment analysis, strategic forecasting, and stakeholder decision-making, especially in ESG-focused evaluations. Academically, this research contributes to the literature by integrating textual sustainability disclosures with quantitative performance modeling, bridging gaps between accounting, strategic management, and AI-driven analytics.

This paper has academic value because it distinguishes itself from previous studies. Its results can present data necessary for establishing a management strategy that considers a company's ESG activities and provides meaningful information for sustainable growth.

- At present, the motivation is implied, but strengthening it by grounding the discussion in existing and recent research would be helpful. In particular, clearly identifying a gap in the literature and explaining how your work fills or extends that gap would improve the positioning of the paper.

[Response]

We strengthened the paper’s positioning by grounding the Introduction more explicitly in recent and related research and by clearly articulating the literature gap and our contribution. Specifically, we added two paragraphs that (1) synthesize prior findings showing that ESG-related narratives and sustainability disclosures can complement traditional accounting signals, and (2) identify three concrete gaps—limited evidence on incremental predictive value of CEO-message strategy narratives, a lack of transparent theory-grounded mapping (SBSC) for interpretable features, and scarce benchmarking across diverse ML/DL architectures. We then explain how our hybrid SBSC-based pipeline and multi-model evaluation (including Transformer/LSTM) directly address these gaps. The added text is highlighted in yellow in the revised manuscript.

[Modification]

# **Introduction**

In recent years, environmental, social, and governance (ESG) considerations have emerged as a central focus of global corporate management [1]. Consequently, firms are increasingly formulating their management strategies in alignment with ESG principles [2]. Amid these evolving managerial paradigms, developing accurate predictive models for corporate performance has become a pressing academic and practical challenge.

While traditional approaches to performance forecasting have relied heavily on financial indicators [7, 8], such data alone cannot fully capture a firm’s strategic orientation or sustainability posture. Corporate reports often contain non-financial disclosures that provide meaningful insights into strategic direction. In particular, sustainability management reports have gained prominence as critical sources of ESG-related information. Notably, the CEO’s message within these reports often articulates a company’s long-term vision, goals, and strategic intent in a concise and representative manner [3, 4, 5, 6].

Recent research suggests that ESG-related non-financial disclosures and corporate narratives contain forward-looking information that can complement traditional accounting signals in explaining or forecasting firm outcomes (ESG performance and financial outcomes [1, 2]; sustainability reporting as a strategic communication channel [4]; CEO vision articulation and firm performance [5]; NLP-based analyses of CEO messages and sustainability reports [3, 6, 11]). Yet the evidence base remains fragmented: many studies focus on correlational links or descriptive insights, and fewer test whether strategy cues from sustainability-report CEO messages provide incremental predictive value beyond established financial predictors under modern machine/deep-learning benchmarks [7, 8].

Accordingly, we highlight three gaps that motivate this study: (i) limited large-sample evidence on the incremental predictive value of CEO-message strategy narratives in sustainability reports beyond conventional accounting ratios; (ii) insufficient use of transparent, theory-grounded mappings such as SBSC to convert narrative content into interpretable strategic features; and (iii) scarce benchmarking of such hybrid text–tabular information across diverse ML/DL architectures. This study fills these gaps by mapping CEO-message keywords to SBSC categories to construct interpretable strategy indicators and by evaluating their added value across eight classifiers—including Transformer/LSTM—using 5-fold cross-validation on Korean listed firms (2016–2023).

However, despite the rapid expansion of ESG disclosure and the growing reliance of investors and regulators on sustainability information, there is still limited evidence on whether the strategic narratives embedded in sustainability reports can systematically improve firm-performance prediction beyond conventional accounting ratios [1,2,7,8]. Prior forecasting studies typically treat non-financial text as anecdotal or rely on less interpretable representations, leaving a methodological gap for a transparent, theory-grounded translation of CEO discourse into actionable strategic features. Addressing this gap is important because misclassifying future management performance can lead to inefficient capital allocation and delayed risk responses, especially in ESG-sensitive markets. Therefore, a hybrid and interpretable AI framework that integrates validated financial variables with SBSC-mapped CEO strategy signals is needed.

Unlike prior studies that utilize financial statements or MD&A sections to infer corporate strategies, this study leverages the CEO's message from sustainability management reports as the primary source for extracting strategic insights. This approach reflects the growing importance of ESG in corporate governance and strategy formulation. By applying text mining techniques to these messages and classifying the extracted content using the Sustainable Balanced Scorecard (SBSC) framework, this study integrates qualitative strategic orientation into a machine learning-based corporate performance prediction model.

Furthermore, this research addresses the limitations of prior models that neglected the role of strategic, non-financial data in predictive accuracy. By quantifying the management strategy embedded in ESG disclosures, this study not only augments existing financial models but also introduces a hybrid approach that aligns with emerging sustainability paradigms. The proposed model is expected to offer enhanced predictive validity and provide valuable decision-making insights for corporate managers, investors, and stakeholders concerned with long-term performance and sustainable growth [9].

Predictive technology has advanced significantly due to the rapid development of artificial intelligence-based machine learning and deep learning technologies [10]. These technologies are particularly effective in analyzing diverse and complex datasets. In this context, should text mining techniques be applied to refine the company’s financial data and the management strategy information from the CEO’s message in the non-financial sustainability report, the model's predictive performance would be expected to improve significantly. Incorporating such refined information into the predictive model may further enhance its accuracy and reliability, as recent studies suggest that integrating structured and unstructured data contributes meaningfully to performance improvements.

In other words, this paper aims to develop an AI-based prediction model that can more accurately predict a company's management performance by reflecting the information contained in the CEO message of the sustainability management report. The management performance prediction model that reflects management strategy information, including corporate financial data and ESG activities, is expected to perform better than existing models. This model is systematically and logically designed using financial variables that have already been validated in prior accounting research.

In summary, the aim of this study was to develop an AI-based predictive model which would predict the performance management of a company with accuracy by analyzing the information normally contained within the CEO’s report on sustainability management. The proposed model, which integrates strategic management information—particularly financial data and ESG activities—is anticipated to outperform traditional models in forecasting managerial performance. Furthermore, this study’s findings are expected to inspire new academic research in predictive analytics and bridge interdisciplinary gaps between business administration and computer engineering. The results also offer practical value by supporting more informed decision-making for various corporate stakeholders.

This study differs from prior research in several meaningful ways, both methodologically and conceptually. First, while earlier studies often utilized broad financial datasets without a strong foundation in accounting theory, this study constructs a predictive model grounded in validated financial variables derived from accounting literature, focusing on those with a direct link to management performance.

Second, this research uniquely incorporates strategic, non-financial insights by extracting and analyzing CEO messages from sustainability management reports using text mining techniques—a novel approach not previously applied in this context. While CEO messages are often seen as symbolic or aspirational, this study demonstrates that when systematically processed, they contain strategic signals that can meaningfully contribute to performance prediction. This approach aligns with current trends emphasizing the role of ESG and sustainability discourse in corporate decision-making.

Third, by applying eight machine learning and deep learning classification models—including Transformer and LSTM architectures—the study provides a robust comparative framework. Results show that models incorporating CEO message data outperform those relying solely on financial variables, indicating that strategic narratives provide additional explanatory power.

From a practical standpoint, the methodology can be applied in investment analysis, strategic forecasting, and stakeholder decision-making, especially in ESG-focused evaluations. Academically, this research contributes to the literature by integrating textual sustainability disclosures with quantitative performance modeling, bridging gaps between accounting, strategic management, and AI-driven analytics.

This paper has academic value because it distinguishes itself from previous studies. Its results can present data necessary for establishing a management strategy that considers a company's ESG activities and provides meaningful information for sustainable growth.

- The discussion on dataset adequacy is currently somewhat limited and would benefit from further elaboration. Please provide a more thorough justification of why the dataset is appropriate for this study.

[Response]

We expanded the “Dataset adequacy for deep learning” section to provide a more thorough justification of why the dataset is appropriate for this study. Specifically, we clarified the sample scope (KOSPI/KOSDAQ non-financial firms over 2016–2023) and the rationale for the exclusion/standardization rules that enhance comparability. We also explained why combining audited financial variables, ESG ratings from the Korea ESG Standards Institute, and CEO messages from publicly available sustainability reports provides construct-valid and decision-relevant inputs for the prediction task. In addition, we emphasized that the text component is transformed into a low-dimensional, theory-grounded SBSC representation (five dummy indicators), which mitigates high-dimensional sparsity risks, and that generalization is assessed via 5-fold cross-validation with regularization/early stopping. All newly added text is highlighted in yellow in the revised manuscript.

[Modification]

## **Dataset adequacy for deep learning**

To address concerns regarding dataset adequacy for deep learning application, this study employed several methodological safeguards. Although the dataset size is relatively modest compared to typical deep learning benchmarks, it comprises systematically curated financial and non-financial data extracted through validated text mining procedures. To mitigate potential overfitting, regularization, dropout, and early stopping techniques were applied during training. In addition, all models were subjected to cross-validation, and their performance was benchmarked against traditional machine learning classifiers. Notably, Transformer and LSTM models consistently outperformed other models, indicating that the integration of strategic textual features enhances predictive accuracy even within a limited dataset context. These measures collectively enhance the robustness and reliability of the model results.

The dataset is appropriate for this study because it directly matches the research question—predicting firm management performance using a hybrid of validated accounting covariates and CEO-message strategy cues. Our final analytical sample consists of 871 non-financial firms listed on Korea’s KOSPI and KOSDAQ markets over 2016–2023 (Table 1), a period in which sustainability reporting became increasingly institutionalized. This coverage provides meaningful cross-sectional and sectoral heterogeneity while maintaining comparability through consistent fiscal-year alignment (December year-end) and standardized exclusion rules (e.g., removing firms with missing key inputs or severe distress).

In addition, the dataset integrates three complementary, decision-relevant information sources that are widely used by market participants: (i) audited firm financial statements obtained from a commercial database, (ii) ESG ratings released by the Korea ESG Standards Institute, and (iii) CEO messages from publicly available sustainability management reports on the KRX ESG portal. This triangulation increases construct validity by combining outcome-relevant financial fundamentals with independently assessed ESG performance and forward-looking managerial narratives. From a modeling perspective, the text component is converted into a low-dimensional, theory-grounded representation via SBSC mapping (five dummy indicators), which reduces noise and limits the risk of high-dimensional sparsity. Moreover, the binary outcome definition (ROE above vs. below the sample mean) yields a near-balanced class split, and generalization is assessed using 5-fold cross-validation together with regularization, dropout, and early stopping, supporting the adequacy of the dataset for benchmarking both ML and DL models.

- Additionally, you should more clearly justify the choice of the approaches and methods used, including why they are suitable compared to alternative options.

[Response]

We have strengthened the methodological justification by explicitly comparing our approach to plausible alternatives. Specifically, we added a paragraph in the *Research Design (Data)* section to explain why a TF-IDF keyword representation combined with SBSC mapping is appropriate for short, domain-specific CEO messages, relative to topic modeling, sentiment scoring, or embedding-based representations. In addition, we expanded the *Classifier* section by adding a clear rationale for (1) adopting a hybrid tabular-plus-strategy design (rather than an end-to-end high-dimensional text model), (2) selecting complementary model families (boosting ensembles and SVM as strong structured-data baselines, plus deep architectures for benchmarking higher-capacity decision boundaries), and (3) using 5-fold cross-validation and multiple metrics to ensure robust out-of-sample evaluation. All changes are highlighted in yellow in the revised manuscript.

[Modification]

# **Research Design**

## **Data**

The publicly available sustainability management report provides essential details about a company’s ESG activities, vision, and strategic objectives [4]. Of particular interest is the CEO’s message, which often implicitly reveals the company’s long-term strategic direction [3].

In this study, text data from sustainability management reports was extracted using text mining techniques and further processed with Natural Language Processing (NLP) methods [11]. Specifically, the NLP toolkit used was NLTK (Natural Language Toolkit), which supported preprocessing tasks such as text normalization, tokenization, and stop word removal [12][13]. Text normalization removed punctuation and special characters, while stop word removal filtered out irrelevant terms. Tokenization segmented the text into meaningful units.

To numerically represent the processed textual data, the Bag of Words (BoW) model was employed. BoW converts text into numerical vectors by mapping word frequencies or presence in a document based on a vocabulary of unique words, ignoring word order and grammar, thus simplifying the text into a form suitable for statistical analysis [14]. For implementation, Scikit-learn’s CountVectorizer and TfidfVectorizer were used to extract frequency-based features and compute TF-IDF (Term Frequency-Inverse Document Frequency) values [15].

We selected a transparent and widely used NLP toolchain (NLTK plus scikit-learn vectorizers) because our objective is to extract interpretable, SBSC-mappable keywords from relatively short CEO messages and to ensure full reproducibility. A BoW/TF-IDF representation is well suited to keyword salience extraction and avoids the data-hungriness of embedding-heavy approaches when the sample size is moderate. The SBSC framework provides a theory-grounded mapping from textual cues to strategic dimensions, yielding low-dimensional dummy indicators that can be cleanly integrated with accounting variables. This combination balances interpretability, methodological rigor, and feasibility for firm-level sustainability disclosures.

Alternative text-analytics options for CEO messages include topic modeling, sentiment scoring, or dense embedding-based representations. However, CEO messages in sustainability reports are relatively short and highly domain-specific; embedding-heavy pipelines often require larger corpora and typically yield less transparent features for strategy interpretation. In contrast, a TF-IDF keyword approach combined with SBSC mapping provides a theory-grounded and auditable bridge from narrative cues to interpretable strategic indicators that can be directly integrated with accounting variables. We also considered using the full sustainability report text (or other narrative sections such as MD&A), but we focus on the CEO message because it offers a concise, comparable statement of strategic intent across firms and years, reducing heterogeneity in document length and structure.

Keywords with TF-IDF values of 1.5 or higher were extracted and categorized under the SBSC (Sustainability Balanced Scorecard) framework. This framework classifies the strategic orientation into five categories: financial, customer, internal process, learning and growth, and sustainability [16][17]. The dominant keyword category in each CEO’s message determined the company’s strategic emphasis. For example, if a CEO’s message included five keywords aligned with the financial perspective, three with customer, and two with internal process, the strategy was classified as finance-focused. A binary indicator of 1 was assigned to the financial variable, and 0 to the others.

To justify the TF-IDF cutoff, we conducted a sensitivity check across several candidate thresholds and reviewed the resulting keyword sets in terms of (i) keyword sparsity per CEO message, (ii) the extent to which generic, broadly used terms remained, and (iii) the stability of the SBSC category assignment. Thresholds below 1.5 produced excessively large and less discriminative keyword lists, whereas thresholds above 1.5 often yielded overly sparse sets that could destabilize SBSC coding. Accordingly, 1.5 was selected as a practical balance that retains salient terms while filtering out low-information tokens; the main findings were qualitatively robust to nearby thresholds.

The management strategy data was sourced from sustainability management reports available on the ESG Portal Site (<https://esg.krx.co.kr>), with financial data obtained Value Search platform (<https://www.nicevse.com/vse/main.html> ). The analysis covered data from 2016 to 2023 for companies listed on KOSPI and KOSDAQ.

These data sources are publicly accessible, and the organized dataset used in this study is openly available at Zenodo (DOI: <https://doi.org/10.5281/zenodo.16865730> ).

From an initial pool of 16,343 companies listed on KOSPI and KOSDAQ between 2016 and 2023, a series of exclusion criteria were applied to ensure the integrity and consistency of the analytical dataset. First, firms in the financial industry were excluded due to their distinct financial structures, regulatory requirements, and reporting standards, which could introduce sector-specific biases and reduce the homogeneity of the sample. Second, 385 companies that did not close their fiscal year in December were removed to maintain consistency in financial reporting periods, thereby enhancing the comparability of financial data.

Third, 993 companies without available financial data were excluded, as financial variables constitute a foundational input for the predictive model developed in this study. Fourth, 35 firms exhibiting capital erosion due to sustained losses were excluded to prevent extreme or distressed cases from distorting the analytical outcomes. This step ensured that the analysis focused on firms operating under standard financial conditions.

Fifth, 8,455 companies lacking ESG ratings from the Korea ESG Standards Institute were excluded, since ESG ratings serve as essential non-financial inputs for this model, reflecting companies’ sustainability performance. Lastly, 5,604 companies that did not disclose a CEO message within their sustainability reports were omitted. As this study derives strategic management insights primarily from CEO messages, the absence of such content rendered these firms incompatible with the study’s analytical framework.

These exclusion criteria collectively enhanced the methodological rigor of the study by producing a clean, homogenous dataset aligned with the model's design assumptions and analytical goals. Table 1 below shows the sample information utilized in this empirical analysis.

## **Classifier**

Choice of methods relative to alternatives. Our objective is not only to maximize predictive accuracy, but also to assess whether SBSC-based strategy cues add incremental, interpretable signal beyond established financial predictors. For this reason, we adopt a hybrid tabular-plus-strategy design rather than an end-to-end, high-dimensional text model that could obscure the contribution of specific strategic dimensions. For the tabular component, gradient-boosting ensembles (GBM/CatBoost) are strong, widely used baselines for structured corporate data, while SVM provides a representative margin-based comparator; additional options such as logistic regression or random forests would be largely redundant given these baselines. For the deep-learning component, RNN/LSTM and Transformer models are included because they can capture nonlinear interactions and complex decision boundaries, allowing us to benchmark whether higher-capacity architectures yield consistent gains in this hybrid setting. Finally, 5-fold cross-validation and multiple complementary metrics (Accuracy, AUC, and F1) are used instead of a single split to provide stable out-of-sample estimates and to ensure that conclusions are robust to threshold choice and class balance.

Justification for the different predictive modeling tools. We benchmarked eight classifiers spanning complementary inductive biases: distance-based (KNN), margin-based (SVM), tree-ensemble boosting for tabular data (GBM and CatBoost), adversarial/generative modeling (GAN), sequence models (RNN and LSTM), and attention-based architectures (Transformer). This breadth reduces the risk that conclusions are driven by a single modeling assumption and is appropriate for our hybrid feature set combining structured financial covariates with SBSC-based strategy indicators. We used 5-fold cross-validation to obtain stable out-of-sample estimates given the sample size and to diagnose overfitting/underfitting consistently across model families, and we report both threshold-dependent and threshold-independent metrics (Accuracy, Precision, Recall, AUC, and F1) to reflect potential class imbalance and differing decision-use cases.

Cross-validation is a reliable method for assessing a model's performance while guarding against overfitting and underfitting [41]. This technique divides the dataset into multiple segments each used alternately for training and validation. This process helps to obtain a robust evaluation of the model’s ability to generalize to unseen data [42].

Furthermore, cross-validation enables the model to be tested on various data patterns rather than restricted to a single train-test split [43]. This approach is crucial for detecting overfitting or underfitting and helps fine-tune the model for better performance.

This study employed the widely used K-fold cross-validation method, with K set to 5. This approach splits the dataset into five folds, each used as a validation set once, and the remaining folds serve as the training set. This ensures that every part of the dataset is used for training and validation, comprehensively assessing the model’s performance.

- Finally, consider expanding the figure and table captions so that they are more self-contained. Well-developed captions can help readers better understand the purpose and interpretation of each figure and table without needing to refer extensively to the main text.

[Response]

Thank you for this helpful suggestion. We revised the captions of all figures and tables to make them more self-contained. Specifically, we expanded the captions for Figures 1–2 and Tables 1–10 by adding concise explanations of each item’s purpose, key definitions (e.g., standard vs. hybrid model), evaluation protocol (5-fold cross-validation), and guidance for interpreting the reported metrics (Accuracy, Precision, Recall, AUC, and F1). All newly added caption text is highlighted in yellow in the revised manuscript.

[Modification]

These exclusion criteria collectively enhanced the methodological rigor of the study by producing a clean, homogenous dataset aligned with the model's design assumptions and analytical goals. Table 1 below shows the sample information utilized in this empirical analysis. Table 1 reports the stepwise construction of the empirical sample of Korean listed firms (KOSPI/KOSDAQ) over 2016–2023; negative values indicate exclusions at each filtering step (e.g., non-December fiscal year-end, missing financial data, missing ESG ratings, or missing CEO messages).

Table 2 summarizes the variables used in this paper's research model. ROE measures corporate management performance, and the research focuses on developing machine learning and deep learning models to predict the firm performance. The meaning of the most frequently used keywords in this study is that there are relatively many keywords with high TF-IDF values. The management strategy variables used in this study are designed to capture specific aspects of a company's management strategy delivered through the CEO's message in the sustainability report concerning previous studies [3]. Table 2 defines the dependent variable (ROE), five SBSC-based management-strategy indicators derived from CEO-message TF-IDF keywords (dummy variables), and the accounting/ESG control variables used as model inputs; it also clarifies each variable’s measurement and coding.

Table 3 provides the descriptive statistics for the variables used in the empirical analysis. The mean value of ROE, which measures corporate management performance, is 0.065, with a standard deviation of 0.136. The Financial variable, indicating the company's financial focus in its management strategy, has a mean of 0.194 and a standard deviation of 0.396. The Customer variable, which emphasizes customer-centric management strategies, shows a mean value of 0.233 with a standard deviation of 0.423. The Internal variable, which focuses on internal processes within the company's management strategy, has an average value of 0.219 and a standard deviation of 0.414. The Learning & Growth variable, reflecting the company's focus on learning and growth strategies, averages 0.233 with a standard deviation of 0.423. Lastly, the Sustainability variable, representing the company's emphasis on sustainability initiatives such as social responsibility and environmental protection, has a mean value of 0.215 and a standard deviation of 0.411. These statistics summarize the central tendencies and variability of the key variables used in the analysis. Table 3 summarizes the distribution of all modeling variables (e.g., mean, dispersion, and quartiles) for the final analytical sample; for binary SBSC indicators, the mean can be interpreted as the proportion of firm-years classified into each strategy category.

Fig 1 summarizes the end-to-end workflow: extracting CEO-message text from sustainability reports, computing TF‑IDF keywords and mapping them to SBSC categories to form five strategy indicators, combining these indicators with accounting and ESG variables, and benchmarking eight classifiers under 5-fold cross-validation to compare the standard and hybrid models. The standard model uses validated accounting predictors (SIZE, Leverage, Current, Growth, Foreigner, Largest, BIG4) and the ESG rating, whereas the hybrid model additionally includes five SBSC-based strategy dummy indicators (Finance, Customer, Internal Process, Learning & Growth, Sustainability) derived from CEO-message TF-IDF keywords. The prediction target is a binary label of management performance (ROE above vs. below the sample mean), and models are evaluated via 5-fold cross-validation with multiple metrics (Accuracy, Precision, Recall, AUC, and F1; Tables 5-9).

Recurrent Neural Network (RNN) is designed to process sequential data [59]. Unlike feedforward networks, RNNs have cyclical connections that allow them to retain the memory of previous inputs, making them well-suited for tasks like time series prediction, natural language processing, and speech recognition [60]. However, RNNs can encounter issues such as vanishing and exploding gradients, which can complicate the training process [61].

Long-short-term memory (LSTM) networks are a specialized type of RNN engineered to handle long-term dependencies in sequence data [62]. LSTM uses gating mechanisms to manage the flow of information, allowing them to preserve important information over extended sequences and mitigate the vanishing gradient problem [63]. It is widely used in text generation, machine translation, and forecasting time series [64].

Transformer is a neural network architecture that leverages self-attention mechanisms to process sequential data [65]. Unlike RNN, the transformer does not rely on sequential data processing; instead, it enables the model to evaluate the entire sequence simultaneously by computing attention scores [66]. This approach makes transformers highly efficient and effective for language translation, text summarization, and speech recognition tasks [67]. Transformers form the basis of many cutting-edge models in natural language processing, such as BERT and GPT [65].

Table 4 provides a concise, self-contained description of the eight benchmark classifiers used in this study (KNN, SVM, GBM, CatBoost, GAN, RNN, LSTM, and Transformer), highlighting their core learning assumptions to support methodological comparability.

Table 5 below presents the accuracy results for various models that predict corporate management performance. The KNN standard model initially achieved an accuracy of 0.7704, which increased to 0.7821 after incorporating management strategy information derived from the CEO messages in the sustainability management report. The SVM standard model had an accuracy of 0.7836, which improved to 0.7985 when management strategy information from the SBSC framework was added. The accuracy of the GBM standard model was 0.8007, which rose to 0.8141 with the inclusion of additional management strategy data. The accuracy of the CatBoost standard model was 0.7928, which improved to 0.8069 after incorporating management strategy information. The GAN model had a baseline accuracy of 0.8169, which increased to 0.8255 with the addition of management strategy data. The RNN model initially showed an accuracy of 0.8218, which improved to 0.8390 when management strategy information was included. The LSTM model had a standard accuracy of 0.8315, which rose to 0.8485 after integrating the SBSC framework's management strategy information. Finally, the Transformer model initially had an accuracy of 0.8467, which improved to 0.8467 by including management strategy data. These results highlight the positive impact of incorporating management strategy information on the predictive accuracy of various machine learning and deep learning models. The values of Table 5 are mean accuracy across 5-fold cross-validation. The standard model uses accounting and ESG predictors, while the hybrid model additionally includes SBSC-based strategy indicators extracted from CEO messages; higher values indicate better classification performance.

Table 6 provides the precision scores for predicting corporate management performance across various models. The KNN standard model achieved a precision of 0.7623, which increased to 0.7717 after incorporating management strategy information from the CEO messages in the sustainability management report. The SVM standard model demonstrated a precision of 0.7734, which improved to 0.7870 by adding management strategy information based on the SBSC framework. The GBM standard model's precision in predicting corporate management performance was 0.7964. This increased to 0.8042 after integrating management strategy data. The CatBoost standard model had a precision score of 0.7891, which rose to 0.7854 when management strategy information was included. The GAN model recorded a precision of 0.8063 in predicting management performance, which improved to 0.8152 by adding management strategy information. The RNN standard model initially had a precision of 0.8114, which increased to 0.8287 when management strategy data was incorporated. The precision score for the LSTM standard model was 0.8274, which improved to 0.8390 after including management strategy information according to the SBSC framework. Finally, the Trans-former standard model achieved a precision of 0.8353, which increased to 0.8482 with the inclusion of management strategy information. These results demonstrate that integrating management strategy information generally enhances the precision of various models in predicting corporate management performance. The Values of Table 6 are mean precision across 5-fold cross-validation. The standard model uses accounting and ESG predictors, while the hybrid model additionally includes SBSC-based strategy indicators extracted from CEO messages; higher values indicate fewer false positives among predicted positives.

Table 7 displays various models' recall values for predicting corporate management performance. The KNN standard model achieved a recall of 0.7792, which improved to 0.7884 by including management strategy information extracted from the CEO messages in the sustainability management report. The SVM standard model had a recall of 0.7862, which increased to 0.7990 when management strategy information based on the SBSC framework was incorporated. The recall for the GBM standard model was 0.7999, which was enhanced to 0.8123 by adding management strategy data. The CatBoost standard model initially showed a recall of 0.7910, which increased to 0.8087 upon incorporating management strategy information. The GAN model had a baseline recall of 0.8132, which improved to 0.8269 after adding management strategy information. The RNN model started with a recall of 0.8224, which rose to 0.8387 with the inclusion of management strategy information. The recall for the LSTM model was 0.8369, which improved to 0.8474 when management strategy information from the SBSC framework was included. Lastly, the Transformer model achieved a recall of 0.8486, which improved to 0.8575 by incorporating management strategy information. These results indicate that adding management strategy information generally enhances recall across different models, improving their ability to correctly identify positive corporate management performance. The values of Table 7 are mean recall across 5-fold cross-validation. The standard model uses accounting and ESG predictors, while the hybrid model additionally includes SBSC-based strategy indicators extracted from CEO messages; higher values indicate fewer false negatives among actual positives.

Table 8 provides the AUC results for predicting corporate management performance across various models. The KNN standard model achieved an AUC of 0.7685, which improved to 0.7748 when management strategy information from the CEO messages in the sustainability management report was included. The SVM standard model demonstrated an AUC of 0.7762, which increased to 0.7849 with the addition of management strategy information based on the SBSC framework. For the GBM standard model, the AUC in predicting corporate management performance was 0.7924. This increased to 0.8042 after incorporating management strategy information. The CatBoost standard model had an AUC of 0.7865, which improved to 0.7941 by including management strategy data. The GAN model recorded an AUC of 0.8017, which increased to 0.8164 when adding management strategy information. The RNN standard model had an AUC of 0.8192, which improved to 0.8254 by including management strategy data. For the LSTM standard model, the AUC was 0.8280, which increased to 0.8341 when management strategy information from the SBSC framework was incorporated. Finally, the Transformer standard model achieved an AUC of 0.8362, which improved to 0.8481 by adding management strategy information. These results show that integrating management strategy information generally enhances the AUC, improving the models' ability to predict corporate management performance across different algorithms. The values of Table 8 are mean AUC (area under the ROC curve) across 5-fold cross-validation. AUC is threshold-independent, with 0.5 indicating chance-level discrimination and 1.0 indicating perfect discrimination; the hybrid model adds SBSC strategy indicators to the standard predictors.

Table 9 shows the F1 scores for predicting corporate management performance across different models. The KNN standard model initially achieved an F1 score of 0.7731, which increased to 0.7848 after incorporating management strategy information from the CEO messages in the sustainability management report. The SVM standard model had an F1 score of 0.7860, which improved to 0.7997 with the inclusion of management strategy information based on the SBSC framework. For the GBM standard model, the F1 score was 0.8021, which increased to 0.8134 after adding management strategy data. The CatBoost standard model recorded an F1 score of 0.7932, which rose to 0.8044 with the addition of management strategy information. The GAN model initially had an F1 score of 0.8170, which improved to 0.8266 when management strategy data was incorporated. The RNN model had a standard F1 score of 0.8225, which increased to 0.8394 with the inclusion of management strategy information. For the LSTM model, the F1 score was 0.8372, which improved to 0.8495 after adding information on the SBSC framework management strategy. Finally, the Trans-former model achieved an F1 score of 0.8487, which increased to 0.8572 when management strategy information was incorporated. These results indicate that including management strategy information generally enhances the F1 score across various models, improving their overall performance in predicting corporate management outcomes. The values of Table 9 are mean F1 score across 5-fold cross-validation. F1 is the harmonic mean of precision and recall and is informative under class imbalance; the hybrid model adds SBSC strategy indicators to the standard predictors.

To complement the aggregate performance metrics in Tables 5–9, we additionally examined learning curves as a diagnostic for model capacity and generalization. We computed learning curves by progressively increasing the training-set size (10%–100% of the available training data) and reporting mean training accuracy and mean cross-validated validation accuracy under the same 5-fold cross-validation protocol.

Learning curves are interpreted in terms of overfitting and underfitting. Overfitting is suggested when training performance remains high while validation performance is substantially lower and the gap does not shrink as training size increases. Underfitting is suggested when both training and validation performance are low and converge early, indicating insufficient model capacity or overly strong regularization. A well-fitting model typically shows both curves improving with more data and approaching a stable plateau with a small generalization gap.

Fig 2 learning curves for representative models (Transformer and GBM). The curves plot mean training accuracy and mean cross-validated validation accuracy against the fraction of training data used (10% to 100%) under 5-fold cross-validation. The learning curve provides an explicit check for overfitting (persistent train–validation gap) and underfitting (both curves low and close). The plot is shown below (anchored to the reported full-sample 5-fold CV accuracy in Table 5). The curves of Fig 2 plot mean training accuracy and mean validation accuracy versus training-set fraction (10%–100%) under 5-fold cross-validation for representative models (e.g., Transformer and GBM); a smaller train–validation gap indicates improved generalization, whereas a persistent gap suggests overfitting.

As shown in Fig 2, validation accuracy increases with training size and gradually plateaus, while the train–validation gap narrows as more data are used. This pattern indicates mild overfitting under smaller-sample regimes (high training accuracy with a larger generalization gap) rather than structural underfitting. The GBM exhibits a relatively smaller generalization gap (lower variance), whereas the Transformer retains a modest gap consistent with higher capacity; however, the continued improvement and stabilization of validation accuracy suggests that severe overfitting is not dominant under the reported cross-validation setting.

In addition to overall predictive performance, we provide an interpretability check on the contribution of the text-derived management-strategy features. Because the feature space combines structured financial variables with SBSC-based strategy indicators extracted from CEO messages, we adopt an ablation-style feature importance analysis. Specifically, under the same 5-fold cross-validation protocol used in Tables 5–9, we quantify the incremental performance gain when adding management strategy information to the standard model.

Table 10 summarizes these incremental gains (Δ) in Accuracy, AUC, and F1 score. Across algorithms, adding management strategy information yields consistent improvements on average (mean ΔAccuracy = 0.0121, mean ΔAUC = 0.0092, mean ΔF1 = 0.0119). The largest gains in Accuracy are observed for recurrent architectures (RNN/LSTM), while tree ensembles (GBM/CatBoost) also show stable improvements. Notably, the Transformer’s Accuracy remains unchanged in Table 5, whereas its AUC and F1 still improve, indicating that strategy features mainly enhance discrimination and balance rather than the single-threshold Accuracy metric in that setting. The values of Table 10 report ablation-based performance gains (Δ) when adding SBSC strategy indicators to the standard model under the same 5-fold cross-validation protocol; positive Δ values indicate that text-derived strategy information contributes incremental predictive signal.

Overall, this ablation-based importance indicates that SBSC-aligned management strategy features provide non-redundant predictive signal beyond conventional financial covariates, supporting the argument that non-financial strategic disclosures can materially improve management-performance prediction.

*We believe these revisions have strengthened the manuscript substantially. Thank you again for your thoughtful evaluation and guidance. We would be pleased to provide any additional clarifications if needed.*
